# Cortical travelling waves relate to variation in personality traits

**DOI:** 10.1162/IMAG.a.119

**Published:** 2025-08-20

**Authors:** Neil W. Bailey, Luiza Bonfim Pacheco, Luke D. Smillie

**Affiliations:** School of Medicine and Psychology, The Australian National University, Canberra, ACT, Australia; Monarch Research Institute Monarch Mental Health Group, Sydney, New South Wales, Australia; School of Psychological Sciences, Monash University, Melbourne, Victoria, Australia; School Psychological Sciences, The University of Melbourne, Melbourne, Victoria, Australia

**Keywords:** cortical travelling waves, personality traits, compassion, openness, electroencephalography

## Abstract

Personality traits must relate to stable neural processes, yet few robust neural correlates of personality have been discovered. Recent methodological advances enable measurement of cortical travelling waves, which likely underpin information flow between brain regions. Here, we explore whether cortical travelling waves relate to personality traits from the “Big Five” taxonomy. We assessed personality traits and recorded resting electroencephalography (EEG) from 300 participants. We computed travelling wave strength using a 3D fast Fourier transform and explored relationships between alpha travelling waves and personality traits. Trait Agreeableness and Openness/Intellect had significant relationships to travelling waves that passed multiple-comparison controls (*p*_FDR_ = 0.019 and *p*_FDR_ = 0.036 respectively). Agreeableness related to interhemispheric waves travelling from the right hemisphere along central lines (rho = 0.263, *p* < 0.001, *BF10* = 356.350). This relationship was unique to the compassion aspect (t = 3.719, *p* < 0.001) rather than politeness aspect of Agreeableness (t = 0.897, *p* = 0.370). Openness/Intellect related to backwards travelling waves along midline electrodes (rho = 0.197, *p* < 0.001, *BF10* = 13.800), which was confirmed for the Openness aspect (rho = 0.216, *p* < 0.001, *BF10* = 26.444) but not the Intellect aspect (rho = 0.093, *p* = 0.109, *BF10* = 0.344). Greater cortical travelling wave strength from right temporal regions was associated with higher trait compassion, and backwards travelling wave strength along midline electrodes was associated with trait openness. Further research is needed to investigate the mechanistic role of travelling waves in personality traits and other individual differences.

## 1 Introduction

Personality traits are propensities towards particular patterns of emotion, cognition and behaviours that differ between individuals (Wilt & Revelle, 2015). The dominant framework for measuring personality traits is the “Big Five” model (John et al., 2008), which organises personality trait variance using five core dimensions. These five dimensions comprise *agreeableness,* where high scoring individuals are characterised by compassion, politeness, and interpersonal warmth, *neuroticism*; where high scoring individuals are characterised by anxiety, depression, and emotional volatility, *conscientiousness*; where high scoring individuals are characterised as organised, hard-working, and goal-oriented, *extraversion*; where high scoring individuals are characterised by sociability, enthusiasm, and assertiveness, *openness/intellect*; where high scoring individuals are characterised as showing imagination, curiosity, and aesthetic sensitivity. These traits are relatively stable over time, partly heritable, have been described across cultures, and are predictive of several life outcomes (Bleidorn et al., 2014; Schmitt et al., 2007; Soto, 2019; Specht et al., 2011).

Given the stability and heritability of personality traits, it is sensible to assume they have some relation to stable neurophysiological patterns. Accordingly, personality neuroscience has investigated relations between personality traits and neural activity in an attempt to understand mechanisms underlying these traits (Allen & DeYoung, 2017; DeYoung et al., 2022). For instance, resting electroencephalography (EEG) has been used to characterise trait-like neural activity (Jach et al., 2020). EEG studies of personality traits commonly examine power within different frequency bands, assumed to reflect the brain’s oscillatory activity (Jach et al., 2020). Oscillations are commonly divided into frequency bands (delta: 1–4 Hz, theta: 4–8 Hz, alpha: 8–13 Hz, beta: 14–25 Hz, gamma: >25 Hz), which have been related to specific cognitive processes (Buzsaki, 2006). For example, alpha activity seems to relate to top-down inhibition of brain regions irrelevant to task demands (Klimesch et al., 2007; Wang et al., 2020). Some studies have found that specific frequencies relate to personality traits, for example, posterior alpha has been shown to negatively correlate with agreeableness (Jach et al., 2020), although a subsequent study failed to replicate this finding (Fruehlinger et al., 2024). Despite the detection of some relationships between personality traits and neural activity, our understanding of the neural correlates of personality is still at an early stage. Furthermore, research in personality neuroscience with robust sample sizes or from meta-analysis typically provide *r*-values of 0.2 or less (e.g., Kuper et al., 2019; Pacheco et al., 2024) and/or do not replicate (Fruehlinger et al., 2024), suggesting that further exploration of neural markers that may underlie personality traits is necessary. Novel approaches are required to provide a more sophisticated understanding of why specific neural activities might correlate with specific personality traits.

Typical analyses of EEG have measured only spatially stationary frequency power, providing information about activity assessed from single electrodes on the scalp. These measures might not capture the multifaceted and complex neural activity related to personality traits (Fruehlinger et al., 2024). A growing area of research suggests that activity measured at each single electrode is driven by cortical travelling waves, where activity spreads across the cortex like waves traversing the ocean (Nunez, 1989; Visser et al., 2017). Furthermore, travelling waves have been shown to explain the majority of the variance in EEG data (Alexander et al., 2019). This suggests that analysis methods that account for both the spatial and temporal dimensions of EEG data, as well as the relationship between the spatial and temporal dimensions, are likely to be more informative than analyses that separate the two dimensions (for example, analyses of spatially stationary frequency power) (Alexander et al., 2019). In support of this suggestion, recent research indicates that cortical travelling alpha waves provide a highly explanatory measure of information flow through the neural hierarchy, with that information flow enabling attention and working memory functions (Alexander et al., 2019; Bailey, Hill, Godfrey, Perera, Hohwy, et al., 2024; Lozano-Soldevilla & VanRullen, 2019; Mohan et al., 2024; Muller et al., 2014; Pang et al., 2020). Thus, cortical travelling alpha waves may provide an index of the engagement of cognitive patterns. Variation in the frequency or strength of engagement in certain cognitive patterns is likely to compose at least a component of the generating factors that underlie different personality traits (DeYoung, 2015; Murdock et al., 2013), suggesting that cortical travelling alpha waves may reflect a neural mechanism worth exploring to explain variation in personality traits.

Furthermore, evidence suggests that cortical travelling alpha waves may be a mechanism by which the brain performs its “predictive processing” functions (Alamia et al., 2023; Tarasi et al., 2025). The predictive processing theory proposes that because our brains can only indirectly receive information (through sensory inputs), they function as a hierarchical Bayesian inference model generator of predictions about expected sensory inputs (Hohwy, 2020; Parr et al., 2022; Sprevak & Smith, 2023). Proponents of the predictive processing theory argue that non-predicted sensory inputs (prediction errors) are passed up the neural hierarchy to update the brain’s predictive model, while predicted inputs are suppressed (Hohwy, 2020; Parr et al., 2022; Sprevak & Smith, 2023). Active inference is then suggested as the mechanism by which the brain acts on its environment (via muscle control) to match the environment to its predictions, enabling goal directed behaviour (Parr et al., 2022). This parsimonious theory is rapidly becoming the dominant paradigm in neuroscience for its elegant explanation that can apply across brain functions to explain behaviours. Research has also indicated that anterior to posterior travelling alpha waves (which show oscillatory phase lags that progressively increase from frontal to posterior electrodes, henceforth referred to as “backwards travelling waves”) index top-down predictions and reflect the direction of attention (Alamia & VanRullen, 2019; Pang et al., 2020; Tarasi et al., 2025). In contrast, posterior to anterior travelling alpha waves (which show phase lags that increase from posterior to frontal electrodes, henceforth referred to as “forwards travelling waves”) have been suggested to provide an index of the bottom-up propagation of prediction errors encoding sensory inputs (Alamia & VanRullen, 2019; Ermentrout & Kleinfeld, 2001; Pang et al., 2020). Thus, the directional patterns of cortical travelling alpha waves provide an indication of information flow patterns through the cortex and are informative of predictive processing functions of the brain. Cortical travelling wave strengths have also been shown to differ in schizophrenia and psychosis (Alamia et al., 2025; Alexander et al., 2009; Castelnovo et al., 2024), as well as in experienced meditators (Bailey, Hill, Godfrey, Perera, Hohwy, et al., 2024), indicating that their patterns are likely to relate to differences in individual traits. Therefore, cortical travelling waves may provide a novel lens through which personality neuroscientists can investigate biological mechanisms underlying personality traits.

Additionally, the explanatory power offered by predictive processing and cortical travelling waves might be informed by evidence for the function of different brain regions. For example, sensory inputs are predominantly processed in posterior brain regions (Aitken et al., 2020; Gordon et al., 2019), while frontal regions are suggested to be responsible for more complex and abstract predictive modelling, including thoughts about past events and future events (Corcoran et al., 2020; Laukkonen & Slagter, 2021). Executive functions like attention control are also suggested to be implemented via application of top-down prediction weightings, indexed by backwards travelling cortical alpha waves (Alamia & VanRullen, 2019; Pang et al., 2020). Individual differences in the balance of these functions might relate to personality traits such as openness/intellect, which has known associations to executive functions (DeYoung et al., 2014; Murdock et al., 2013; Smillie et al., 2016). Alternatively, activity in the right temporoparietal junction has been shown to be associated with the processing of empathy, sympathy, perspective taking, and social cognition (Decety & Lamm, 2007; Gallagher et al., 2000). These processes are relevant to trait agreeableness, so it is plausible that individual variation in information flow from the right temporoparietal junction (which may also be indexed by measuring rightwards travelling cortical alpha waves) could be associated with differences in this trait. However, to our knowledge, no research has explored whether cortical travelling wave patterns relate to any personality traits.

We undertook an exploratory analysis of a large existing dataset, measuring alpha waves travelling forwards, backwards, and laterally across the scalp and relating these to the Big Five personality traits. This may help identify neural mechanisms underpinning personality and offer greater interpretability than associations between other neural activities and personality traits.

## 2 Methods

### 2.1 Participants and procedure

The dataset comprised a total of 300 right-handed participants aged between 18 and 60 years, who were recruited to multiple studies through physical and online notices. The full sample has been described by Pacheco et al. (2024) and a subset of the data was described earlier by Jach et al. (2020). All participants had normal or corrected-to-normal vision and did not report any current mental illness.

After pre-processing the EEG data, 296 participants (176 female, 120 male) provided enough artifact free electrodes and epochs for inclusion in the final analysis (four participants were excluded due to an excessive number of bad electrodes, with the details of these exclusion criteria provided in the Procedure section). The mean age of the participants for the final sample was 23.108 (SD = 6.275).

Ethical approval for the study was provided by the Human Research Ethics Committee of the University of Melbourne (ID 1954069). All participants provided written informed consent prior to participation in the study.

Prior to the EEG recording and task completion, participants provided demographic information and completed the self-report personality questionnaire described below via an online *Qualtrics*^TM^ survey. Demographics included age, sex and hand preference.

### 2.2 Personality assessment

All participants completed the Big Five Aspects Scales (DeYoung et al., 2007), which comprises 100 items to assess the Big Five at two levels of abstraction: The *domain* level comprises openness/intellect, neuroticism, agreeableness, conscientiousness, and extraversion (20 items each). Each of these domains divides into two *aspect* level traits; the ten aspects are openness, intellect, volatility, withdrawal, politeness, compassion, orderliness, industriousness, assertiveness, and enthusiasm (10 items each). Each item consists of a brief descriptive phrase (e.g., for extraversion: *make friends easily*) to which participants respond by indicating their level of agreement that the phrase describes their personality (from 1 = *Strongly Disagree* to 5 = *Strongly Agree*).

### 2.3 EEG acquisition and pre-processing

Sixty-four-channel EEG (BioSemi, Amsterdam, The Netherlands) was recorded from Ag/AgCl electrodes embedded in an elasticised Easy-Cap® and aligned with the extended 10–20 system. Additional electrodes were also placed at the outer canthus and supra-orbit of the left eye, and at the right and left mastoids, although these electrodes were not used in the current analysis. All electrodes’ offsets were within ±40 µV, and EEG data were sampled at 512 Hz with no online bandpass filters. Participants rested with eight interleaved periods of 60 s with either their eyes open or eyes closed; the experimenter instructed participants to switch between eyes open and eyes closed when a visual message signalled the end of each 60 s window. Electrodes were grounded using common mode sense and driven right leg electrodes.

EEG data were pre-processed using the RELAX EEG pre-processing pipeline (Bailey, Biabani, et al., 2023), a toolbox that uses EEGLAB functions (Delorme & Makeig, 2004), with selected application of Fieldtrip functions (Oostenveld et al., 2011). The RELAX cleaning pipeline first bandpass filters the data between 0.25 Hz and 80 Hz, with a notch filter from 47 to 53 Hz, using fourth order Butterworth filters. The pipeline then uses the PREP toolbox to reject bad electrodes (Bigdely-Shamlo et al., 2015), followed by a secondary rejection of electrodes that show outlying data for more than 5% of the recording using moderately aggressive outlier rejection settings, with outliers detected using measures of kurtosis, the probability of the distribution of voltage values within each epoch, absolute voltage thresholds and voltage shifts within an epoch (Bailey, Hill, Godfrey, Perera, Rogasch, et al., 2024), with the limit that no more than 10% of electrodes could be rejected. Outlying periods remaining in the data were then rejected based on the same outlier identification approaches as the channel rejection step.

Following the rejection of bad electrodes, three sequential multi-channel Wiener (MWF) filters were applied as an initial artifact rejection step to reduce muscle artifact, then blinks, then both horizontal eye movement and remaining drift. To clean EEG data with MWF, the first step involves obtaining templates of EEG data periods that are identified as containing artifacts and periods identified as containing only neural activity. MWF then uses a delay embedded matrix of data from all electrodes in combination with the artifact and clean data templates to obtain a model of the spatio-temporal patterns that characterise the artifacts through a minimization of the mean squared error algorithm (Somers et al., 2018). Here, we used a delay embedding period set to 16, which means that the MWF cleaning characterised each artifact by accounting for a total of 33 ms of data at a time (16 samples forwards and backwards from each timepoint). Since the delay embedding characterises the temporal as well as spatial aspects of the artifacts, MWF acts as a spatio-temporal filter. This allows MWF to reduce artifact topographies by taking into account the temporal patterns as well as the spatial patterns of the artifacts (Somers et al., 2018). This has been suggested to effectively clean artifacts while also better preserving neural activity (Somers et al., 2018).

Following the MWF cleaning, PREP’s robust average referencing was applied to the data (Bigdely-Shamlo et al., 2015). Independent component analysis (ICA) was then computed on the data using the fastica algorithm. Artifactual components remaining in the data were identified by the machine-learning algorithm, ICLabel, with the criteria that if ICLabel identified a component as “most likely” to be artifact, it was selected for reduction (Pion-Tonachini et al., 2019). Artifacts from all categories were identified (eye movements/blinks, muscle activity, heart-beat, line noise, and non-specific other artifacts). These artifacts were then reduced using a stationary wavelet transform to characterise the dominant frequencies within each artifact component, which are assumed to reflect the artifact contribution to the signal, and removed from each component before data were reconstructed back into the scalp space (Castellanos & Makarov, 2006). Missing electrodes were interpolated back into the data using spherical spline interpolation (Perrin et al., 1989). This combination of MWF and wavelet enhanced ICA has been shown to effectively clean EEG data of artifacts, while preserving neural signals (Bailey, Biabani, et al., 2023, Bailey, Hill, et al., 2023).

Following cleaning of the continuous data, data were epoched into 1 s periods with a 0.5 s overlap from the eyes open periods of the recording. Previous research has indicated that backwards travelling waves dominate the EEG recording during eyes-closed resting periods, while forwards travelling waves dominate the EEG during eyes-open visual stimulation (Alamia & VanRullen, 2019; Pang et al., 2020). We were interested in whether personality traits might be related to the strengths of both backwards and forwards waves, so we reasoned that eyes-open resting periods (without visual stimulation) were likely to provide the best balance of detection of both backwards and forwards waves. However, we also performed validation analyses of the eyes-closed resting periods to determine if our results for the eyes-open periods were consistent across both resting states (this was the case, as detailed in the Supplementary Materials). Selected electrodes from these epochs were then extracted to enable computation of the cortical travelling waves (electrodes and computation method described in detail below).

### 2.4 Measures

Personality scores were computed by taking the mean response to the 20 items for each domain and the 10 items for each aspect, for a total of 15 scale scores. We also estimated these scores as latent variables (using maximum likelihood estimation), which reduces measurement error (because only the variance shared by all items within a respective scale is represented). As results based on latent variables did not differ substantively from those based on mean scores, we relegate theses analyses to the supplement.

**Fig. 1.**
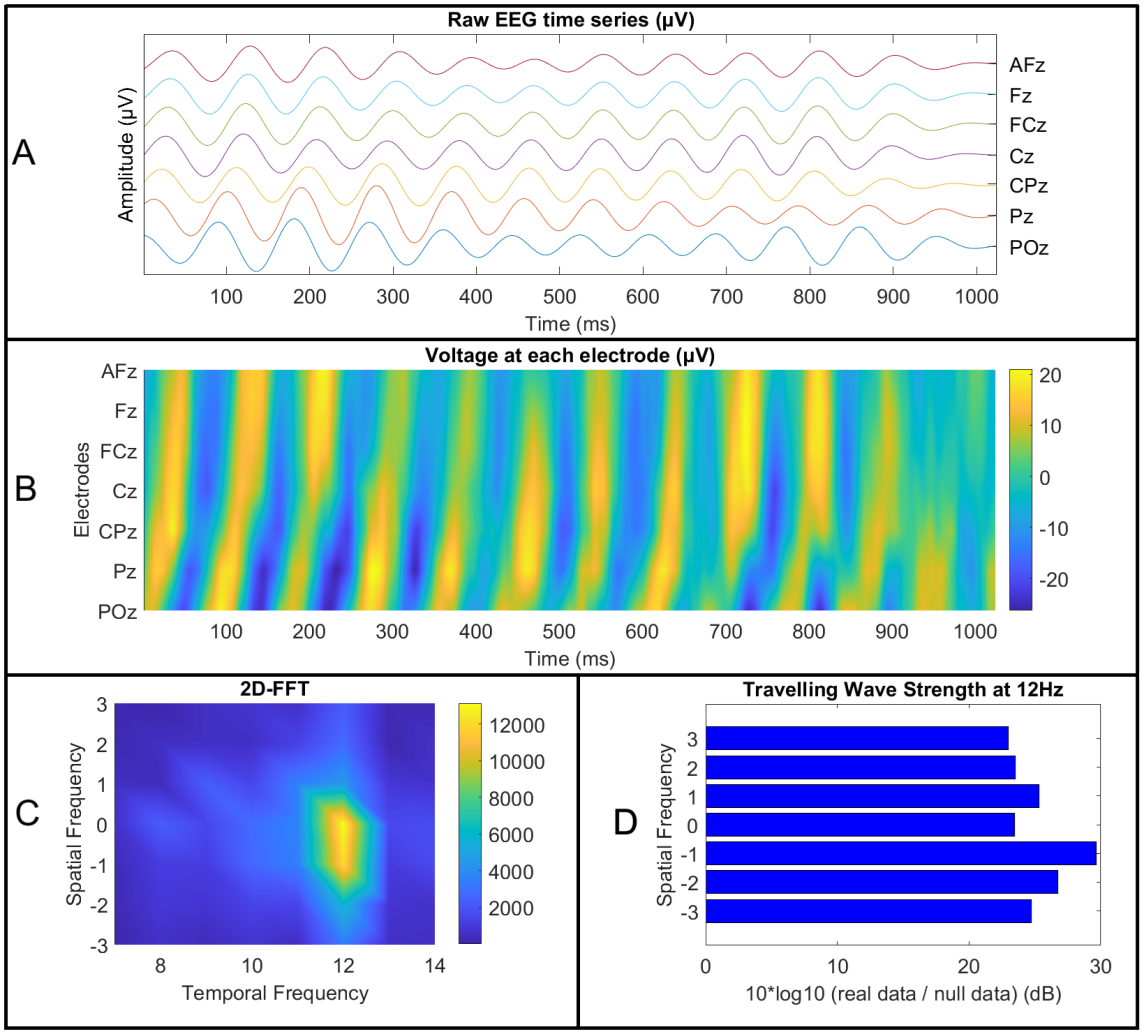
A visual representation of the travelling wave computation method using a 2-dimensional fast Fourier transform (2D-FFT), which can be extrapolated to understand the 3-dimensional fast Fourier transform (3D-FFT) used in our primary analyses. (A, B) First, 1 s epochs were extracted from the continuous electroencephalography (EEG) data after removal of artifacts. The time-series from each electrode were then organised into matrices, with posterior electrodes at the bottom of the matrices and anterior electrodes at the top (and left electrodes on the left side of the 3D matrices, and right electrodes on the right side), as depicted in (A, B) (after applying a bandpass filter in the alpha frequencies of interest to highlight the travelling waves). (C) Each epoch was then used as the input for a 3D-FFT (MATLAB), or 2D-FFT in the case of our post-hoc tests (as depicted above for simplification of the visualisation). This provided a matrix providing power values in each spatial and temporal frequency. Within this matrix, the x-axis provides power values at each temporal frequency, while the y-axis provides power values at each spatial frequency—shown in C with values above 0 reflecting backwards travelling spatial waves (from frontal to posterior electrodes), values below 0 reflecting forwards travelling spatial waves (from posterior to frontal electrodes), and values at 0 reflecting standing waves (waves that do not travel). In our 3D-FFT (not depicted in this figure), the z-axis would reflect lateral travelling waves (from left to right or right to left). (D) Next, to assess the travelling wave strength in each spatial and temporal frequency, we divided the power in each cell in the matrix by the power obtained after randomly shuffling the order of the electrodes in each epoch 100 times, repeating the FFT computations for each shuffle, and averaging the outputs of these null FFTs. We then computed a log10 transform on the output of this division and multiplied this value by 10 to obtain a measure of the strength by which the real travelling wave strength exceeded the permutation-based null travelling wave strength, on the decibel scale (D).

To determine the strength and direction of cortical travelling alpha travelling waves, we expanded the method developed by Alamia and VanRullen (2019) to include 35 electrodes across both lateral and midline locations from both hemisphere (AF7, AF3, AFz, AF4, and AF8, as well as PO7, PO3, POz, PO4, and PO8, and all electrodes between). This allowed the measurement of cortical travelling waves in both forwards and backwards and lateral directions. See Figure 1 for a visual depiction of this methodology. Within each participant, 1 s epochs were extracted from each of the EEG recordings. Following the methods introduced by Alamia and VanRullen (2019), these epochs were extracted every 500 ms within the resting data (so the epochs from the resting data contained 500 ms overlaps with neighbouring epochs). This provided a 3-dimensional matrix for each epoch (forwards and backwards electrode line x lateral electrode line x time). A 3-dimensional fast Fourier transform (3D-FFT) was then applied (using the Matlab function ‘fftn’) to the 3D matrix from each epoch separately. The output matrix of this 3D-FFT provides both temporal frequencies and spatial frequencies, with spatial frequencies represented in the first two axes of the matrix (see Fig. 1). Waves propagating forwards (from occipital to frontal electrodes) are represented in the upper quadrant of the matrix (Fig. 1C), while waves propagating backwards (from frontal to occipital electrodes) are represented in the lower quadrant (Alamia & VanRullen, 2019). Within the 3D version of the matrix, waves propagating from right to left are represented in the left side of the matrix, and waves propagating from left to right are represented in the right side of the matrix (the 3D version of the matrix is not depicted in Fig. 1 due to its complexity).

To ensure these travelling wave values reflected real signals that exceeded simple random patterns in the data, and to control for the potential influence of variation in oscillatory power without variation in travelling wave strength, we performed the same travelling wave computations on 100 surrogate null versions of the data for each epoch. These surrogate null versions were obtained by randomly shuffling the location of all electrodes in the matrix from each epoch separately prior to the computation of the 3D-FFT on this null data (Alamia & VanRullen, 2019). This shuffling of the electrode order made it impossible for the analysis to detect the spatial pattern of travelling waves provided by progressively increasing phase lags between electrodes, but it also preserved the temporal oscillatory pattern, providing a distribution that was matched to the real data for oscillatory power (while reflecting a null distribution for the spatial structure) (Alamia & VanRullen, 2019). First, to ensure that real travelling wave patterns were present in the data (so that our tests of relationships to personality traits were valid), we averaged the real travelling wave and surrogate null travelling wave values between 8–13 Hz, and divided the mean real travelling wave values by the mean surrogate null travelling wave values separately for each travelling wave direction. If there were not travelling waves in the real data, we would expect this computation to provide a value of 1 for each travelling wave direction. We performed one-sample t-tests for each travelling wave direction, testing whether each travelling wave direction significantly differed from 1 across all participants.

Then, to ensure our statistical tests of relationships between personality traits and travelling waves focused on the signal strength of the real travelling waves above the null, we performed a matrix-wise division of the values from the real data within each epoch by the values within the surrogate data for the same epoch, then multiplied the result by 10*log10, as per previous research (Alamia & VanRullen, 2019). This provided a value for each epoch within each participant that reflected the ratio of the strength by which the real travelling waves exceeded the surrogate waves, with values on a log scale (providing values in decibel units [dB]) (Alamia & VanRullen, 2019). Finally, we extracted the 75^th^ percentile travelling wave value within each cell in the matrix, inclusive of backwards and forwards travelling waves at three spatial frequencies, left and right travelling waves at two spatial frequencies, and six temporal frequencies (8 to 13 Hz). For our primary statistical tests, the 75^th^ percentile value was used instead of the mean, as we reasoned that potential differences in travelling wave strength between different personality traits would likely be driven by the engagement of cortical travelling waves, reflecting active neural processing related to a personality trait, more than by the average across all resting neural activity. In support of this rationale, the dominant travelling wave direction has been demonstrated to switch between different time periods, dependent on the engagement of different cognitive processes (Bailey, Hill, Godfrey, Perera, Hohwy, et al., 2024; Pang et al., 2020; Zeng et al., 2024). Based on these previous findings, we suspected that relationships between personality traits and cortical travelling waves would be best revealed by examining periods when cortical travelling waves were activated to engage cognitive processes that might relate to personality traits, thus we focused our analysis on the 75^th^ percentile of cortical travelling wave strength across epochs. This suspicion was validated in our current data, with an exploratory analysis of the correlation strength between personality traits and travelling wave strengths extracted using a range of percentile values indicating higher correlation strengths for percentiles above the median, and correlation strengths progressively diminishing below the median (see Supplementary Materials Fig. S3). However, we note that the significant effects presented in our results section remained significant when using the mean value instead of the 75^th^ percentile, albeit with a reduced effect size (the full results of these tests are reported in our Supplementary Materials).

Finally, an anonymous reviewer of our manuscript queried whether personality traits were linked to stronger travelling waves, or to more common occurrences of travelling waves (factors that could have both influenced our measures). One method to assess how commonly a brain activity pattern occurs is to determine how often the pattern occurs in the real data relative to a distribution of surrogate data where the data have been shuffled to destroy any pattern of interest in the data. However, because even brief periods of travelling waves increase estimates of travelling wave power, essentially all epochs showed travelling wave values significantly above the distribution of surrogate data created by shuffling electrodes between epochs. We, therefore, deemed this method to be inappropriate to assess the rate of travelling wave occurrence. Instead, we drew inspiration from previous research that assessed shifts in the dominant pattern of travelling wave direction by assessing the strength of travelling waves in one direction relative to the strength of travelling waves in the opposite direction (Alamia & VanRullen, 2019). Thus, to assess inter-individual variability in the occurrence of travelling wave patterns, we segmented our data into 500 ms epochs (to provide higher resolution detection of brief engagements of travelling waves), then computed the proportion of epochs that showed stronger travelling waves in the direction that showed an association with a personality trait, relative to the total number of epochs (inclusive of epochs showing both the travelling wave direction of interest and its opposite direction). This provided a measure of how commonly the travelling wave directional pattern occurred in the data (henceforth referred to as the ‘wave direction dominance ratio’). Then, to assess whether the strength of travelling waves was related to personality traits independently of the proportion of their occurrence (henceforth referred to as the ‘directional wave intensity’), we averaged the decibel measure of travelling wave strength above the null values (as per our primary analyses) after restricting the inclusion of epochs to only the epochs that showed a travelling wave strength that exceeded the travelling wave strength in the opposite direction.

### 2.5 Statistical analyses

Analyses were performed using MATLAB (MATLAB, 2023), R (R-Core-Team, 2024), and JASP 0.17.2.1 (Love et al., 2019). The output of our 3D-FFT provided 35 spatial frequencies and 6 temporal frequencies, which could each be correlated against 5 personality traits (and 10 sub-traits). Given the large number of potential multiple statistical tests with likely dependence between different tests, we used cluster-based statistics, which do not assume independence between tests, but rather leverages the likely dependence between tests to increase the power to detect real effects against a null distribution obtained by shuffling individual labels. First, we performed Spearman’s correlations between each output from the 3D-FFT and each personality trait to obtain a single 3D matrix of rho values for each personality trait. Next, we excluded non-significant correlations (*p* > 0.05) from each 3D matrix of rho values. We then computed the maximum sum of rho values from three adjacent cells, across at least two adjacent frequencies and two adjacent spatial cells that passed our initial threshold for each of these 3D matrices (*p* < 0.05). Following this, we performed an absolute transform on the maximum from the 3D rho matrix for each personality trait. Next, we ranked these absolute maximum sum rho values from significant clusters against the absolute maximum sum rho values from clusters that passed our initial threshold obtained from 5000 shuffles of the participant labels. This enabled us to obtain an overall *p*-value for the relationship between each personality trait and the overall cortical travelling wave matrix. Clusters from the real data that provided larger absolute maximum sum values than 95% of the null distribution data were deemed significant at *p* < 0.05. Finally, we submitted the overall *p*-value for each personality trait to an experiment-wise false discovery rate (FDR) multiple statistical test control using the method introduced by Benjamini and Hochberg (1995).

Following our tests using cluster-based statistics to determine whether any personality traits correlated to any cortical travelling waves as measured by the output of our 3D-FFT computation, we performed post-hoc tests of relationships between the personality traits showing effects in our overall analysis using 2D-FFT restricted to the patterns that showed the strongest effects in our 3D-FFT. This enabled us to determine the specific pattern of cortical travelling waves that related to each personality trait (since cluster-based statistics can provide an overall measure of significance, but interpretation of the significance of the location of an effect using cluster-based statistics is not valid). Within these 2D-FFT analyses, we first obtained the maximum travelling wave strength within the temporal frequencies of interest (those showing a significant effect within the cluster analyses of the 3D-FFT), which enabled us to reduce the number of comparisons. Then, we performed the same log10 normalisation as described for the 3D-FFT to obtain the 2D travelling wave strength for each individual in each effect of interest.

We then used Spearman’s correlations to examine relationships between personality scores and the specific travelling wave directions and frequencies that showed significant effects within our cluster analysis of the 3D-FFT, and Bayesian Pearson’s correlations between the same variables to obtain Bayes Factors (BF) enabling us to determine the strength of evidence for the null or alternative hypotheses (*BF10* indicates the probability that the alternative hypothesis is accurate given the data compared to the probability that the null hypothesis is accurate given the data). We assigned a uniform prior probability to all values between -1 and +1, reflecting a lack of prior belief.

## 3 Results

### 3.1 Cortical travelling waves were present in all directions in our data

Our one-sample t-tests showed that values for the real travelling wave data divided by the surrogate travelling wave data for all cells in our 3D matrix and all directions significantly differed from 1 (all *p* < 0.001 and all *p*_FDR_ < 0.001), with all means falling between 18 and 255. This result suggests that the travelling wave signals in our data were on average at least 18 times larger than the surrogate null values. This indicates that the presence of travelling waves in our data, and that our tests of relationships between personality traits and travelling wave analyses were valid. Additionally, in our Supplementary Materials, we have reported additional analyses that confirm the presence of the specific cortical travelling waves that we found to be related to personality traits in our primary analyses.

### 3.2 Cortical travelling waves correlated to agreeableness and openness/intellect

**Fig. 2.**
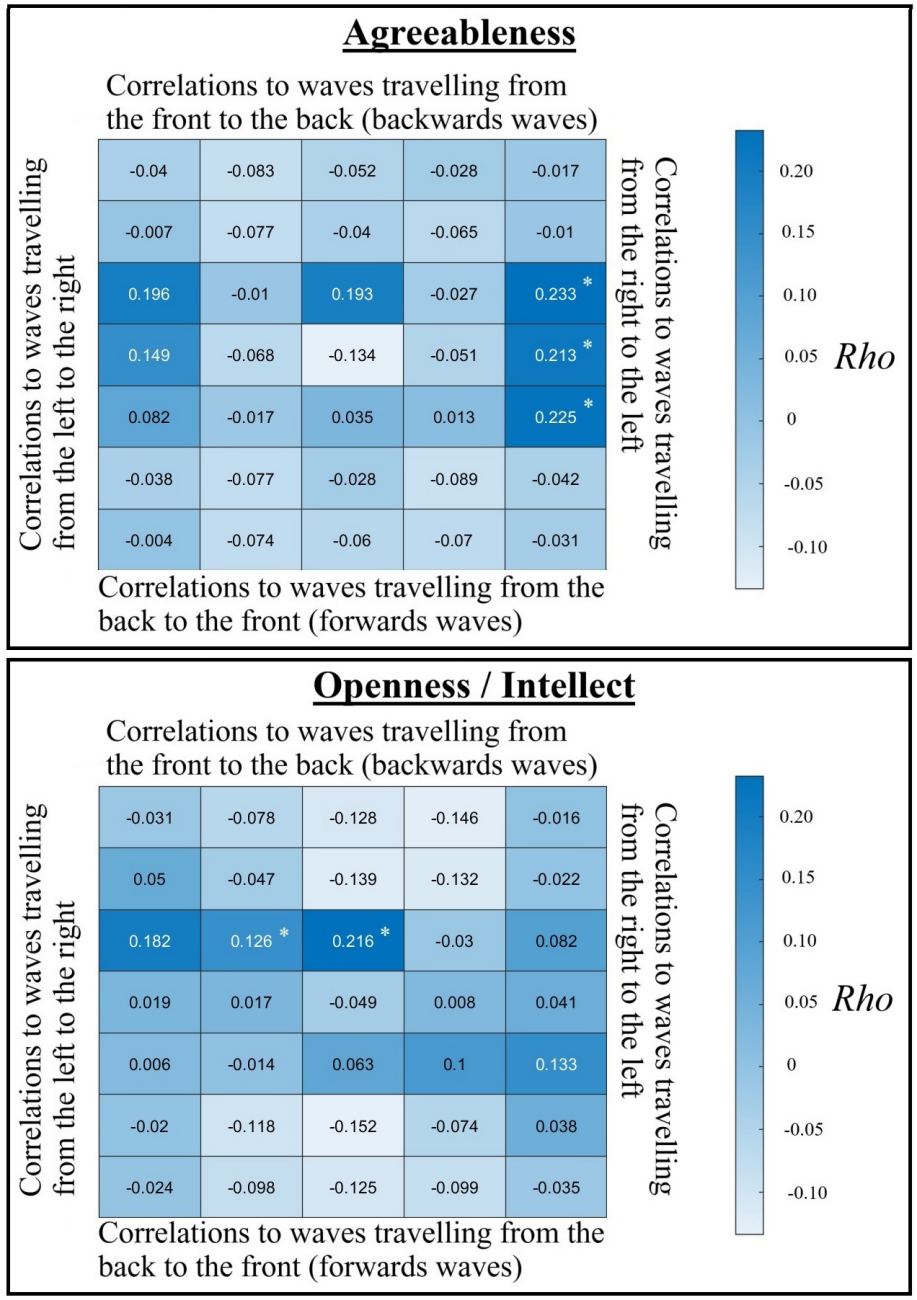
Spearman’s correlations between 3D-FFT travelling wave values at each spatial frequency and direction, and at 10 Hz in the frequency domain, and agreeableness (top) or openness/intellect (bottom). * indicates cells that were involved within a cluster that passed our initial cluster statistical test of relationships between the personality traits and 3D-FFT outcomes (*p*_FDR_ < 0.05), with values in each cell representing travelling waves across the directions that could be measured across the scalp electrodes.

The results of our cluster-based statistical tests indicated that domain-level Agreeableness and Openness/Intellect related to clusters of cortical travelling waves in our eyes-open resting data, with these relationships passing our cluster and Benjamini Hochberg FDR multiple comparison controls (*p* = 0.004, *p*_FDR_ = 0.019 for Agreeableness and *p* = 0.014, *p*_FDR_ = 0.036 for Openness/Intellect, Fig. 2). When using the mean travelling wave strength (instead of the 75^th^ percentile), the same two relationships were present (*p* < 0.001, *p*_FDR_ = 0.003 for Agreeableness and *p* = 0.012, *p*_FDR_ = 0.031 for Openness/Intellect, see Supplementary Fig. S2). None of the other personality traits passed our statistical thresholds: Neuroticism *p* = 0.239, *p*_FDR_ = 0.299, extraversion *p* = 0.039, *p*_FDR_ = 0.064, and conscientiousness *p* = 0.463, *p*_FDR_ = 0.463 (all *p* > 0.05 and all *p*_FDR_ > 0.05 when using the mean travelling wave strength).

The cluster showing the strongest *rho* values for correlations between Agreeableness and cortical travelling waves in our 3D analysis were for waves travelling from the right to the left hemisphere at the highest spatial frequency, along the central, fronto-central, and centroparietal electrode lines, which showed a positive correlation with maximal rho values between 9–10 Hz in the temporal frequency dimension (Fig. 2), but also significant effects from 8–13 Hz within this cluster. After excluding the cluster showing the largest effect, an additional cluster from 8–9 Hz travelling left to right at the maximal spatial frequency and from 8–11 Hz in the temporal frequency dimension also passed our FDR threshold, showing a positive correlation with Agreeableness (*p* = 0.003, *p*_FDR_ = 0.015).

The cluster showing the strongest *rho* values for correlations between Openness/Intellect and cortical travelling waves in our 3D analysis were for backwards travelling waves at the lowest spatial frequency along the midline electrodes with maximal rho values from 10–11 Hz, and significant effects from 9–12 Hz. These clusters showed positive correlations with Openness/Intellect (Fig. 2).

### 3.3 Agreeableness relates to waves travelling from the right hemisphere

**Fig. 3.**
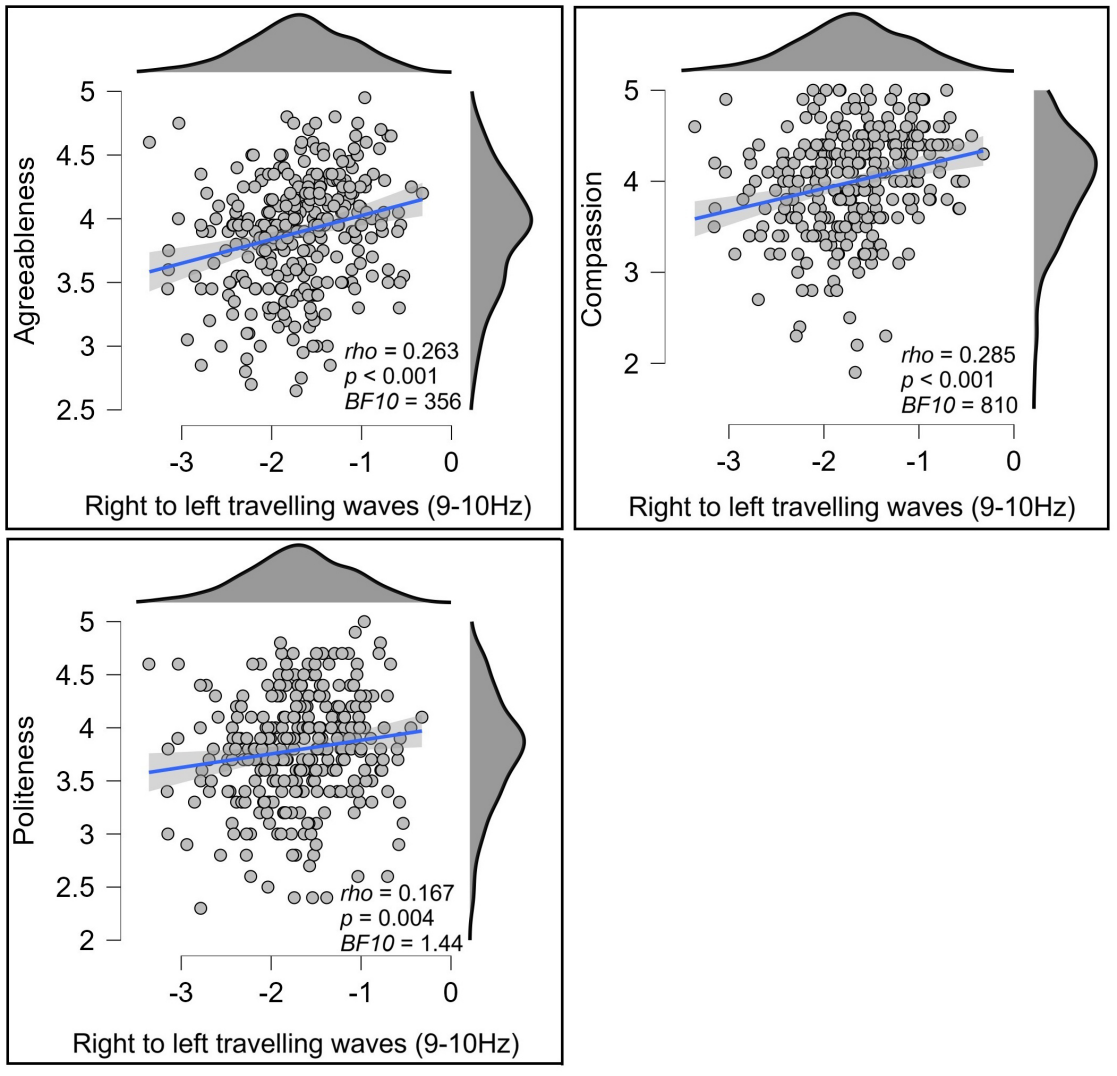
Scatterplots showing the relationship between agreeableness (top left), compassion (top right), or politeness (bottom left) and right to left cortical travelling alpha waves (at 9 to 10 Hz, computed using a 2D-FFT from the central electrode line, units in decibels) within the eyes open resting data. The blue lines indicate the linear regression line with the shaded area indicating the 95% confidence interval for this regression line.

**Fig. 4.**
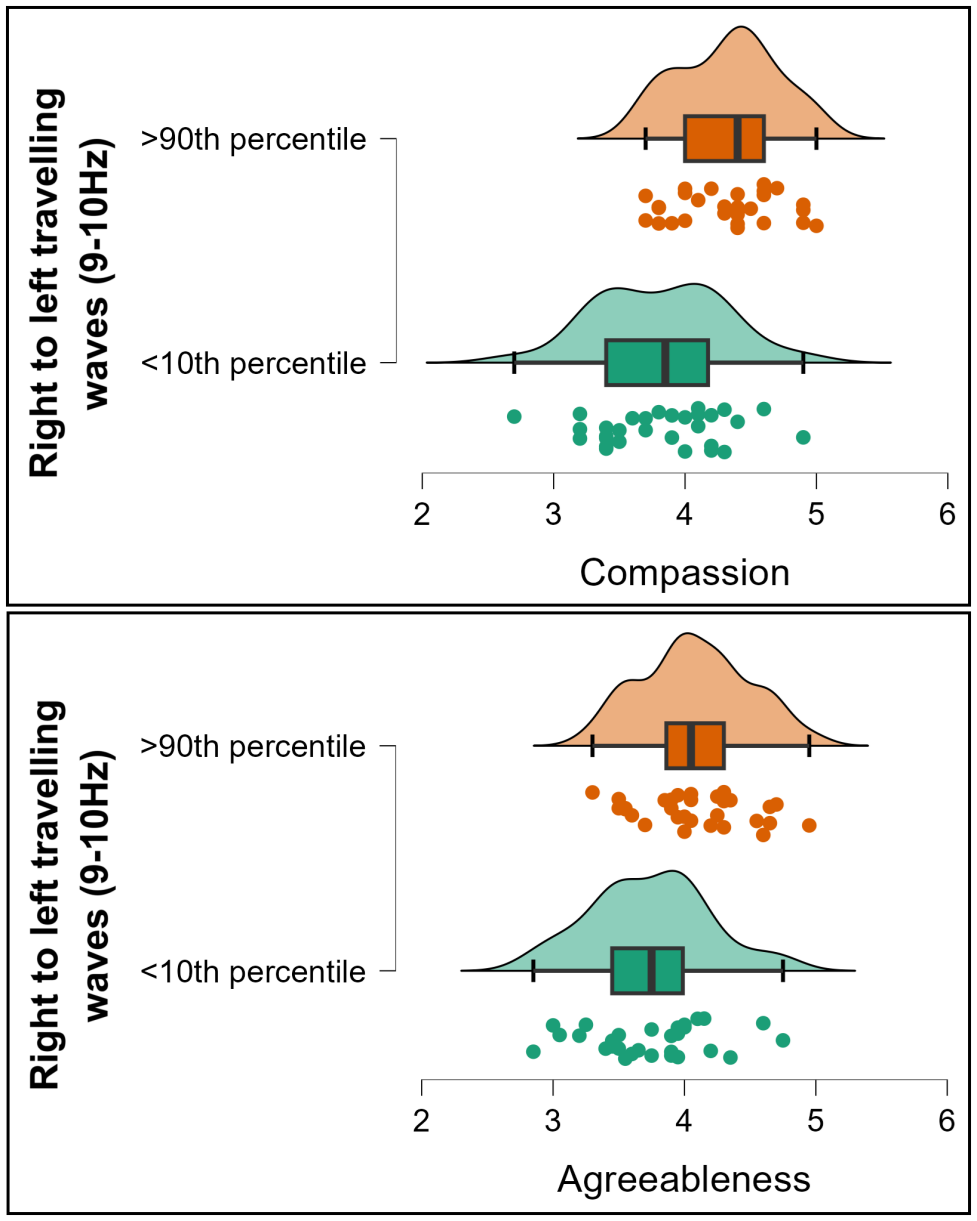
Raincloud plots depicting participants from the top and bottom 10^th^ percentile of right to left travelling wave strengths (9–10 Hz) and their associated scores for compassion (top) and agreeableness (bottom).

Exploration of the significant effects within the 3D-FFT outcomes with post-hoc 2D tests showed that Agreeableness significantly correlated to waves travelling from the right to the left hemisphere along the central electrode lines (T8, C4, Cz, C4, T7) in the 9–10 Hz temporal frequencies (*rho* = 0.263, *p* < 0.001, *BF10* = 356.350, Fig. 3). To test the potential replicability of this result, we randomly split the data into half the sample size 10 times, then tested Spearman’s correlations on each of these reduced samples. All ten reduced samples showed a significant correlation (all *p* < 0.005, mean *p* < 0.001, all *rho* fell between 0.237 and 0.344, mean *rho* = 0.299). Exploring potential relationships between this rightwards travelling wave and the different aspects of agreeableness, we found that the association was appreciably stronger for the compassion aspect of Agreeableness (*rho* = 0.285, *p* < 0.001, *BF10* = 810.438, Fig. 3) than the politeness aspect (*rho* = 0.167, *p* = 0.004, *BF10* = 1.437, Fig. 3), although Hittner et al.’s (2003) test for the difference between dependent correlations with overlapping variables fell just short of formal significance (*z* = 1.912, *p* = 0.056). Given the overlap between compassion and politeness (*rho* = 0.401, *p* < 0.001), a linear regression including compassion and politeness as predictors for the rightwards cortical travelling wave strengths was undertaken. This yielded a significant effect for compassion (*t* = 3.719, *p* < 0.001) but not for politeness (*t* = 0.897, *p* = 0.370), suggesting the effect was specific to compassion. To illustrate the strength of the relationships between cortical travelling waves and agreeableness/compassion, we extracted groups of participants showing the top 10% and bottom 10% of right to left travelling wave strength. The mean compassion score from participants showing the top 10% of right to left travelling wave strength was 13.4% higher than the mean from the bottom 10%, and the mean agreeableness score was 9.2% higher (see Fig. 4).

In addition to the significant rightwards travelling wave cluster in our cluster-based statistical analysis performed on the 3D-FFT outputs, a cluster from 8–9 Hz travelling left to right at the maximal spatial frequency showed a similar positive correlation with Agreeableness. However, the 2D analysis showed weaker support for this relationship, with Bayesian evidence against its existence. We report its full details in the Supplementary Materials for brevity.

Next, to assess the specificity of the rightwards travelling wave effects, and their robustness to different analytical choices, we performed several validation analyses. For brevity, the full report of these results is restricted to our Supplementary Materials, and we only provide a summary here. First, testing the same relationships to rightwards travelling waves during eyes closed resting data (rather than eyes open resting data) showed the same pattern of results and same significant effects, indicating that the patterns are consistent across different resting states. Second, when we tested correlations between the rightward travelling wave and latent variables instead of mean responses, the exact same pattern of results and significant effects emerged. We also tested relationships between cortical travelling waves along the fronto-central, and centroparietal lateral electrode lines and agreeableness (and its aspects). These results showed the exact same pattern of results and significant effects for the centroparietal electrode line (TP7 to TP8), indicating a broad effect from the electrodes over centro/temporo-parietal regions. The analysis of the fronto-central line (FT7 to FT8) showed a similar pattern of results, but with weaker effect sizes.

Next, we tested whether our results were robust to alternative travelling wave strength normalisation procedures. Results of these assessments showed that the relationships to agreeableness were, indeed, driven by variability in actual rightwards travelling wave strength. Positive and significant correlations were still obtained in tests of data that were normalised against mean alpha power across lateral electrodes obtained using a 1D FFT, which controls for variations in alpha power without being influenced by patterns in the null shuffle data (Zeng et al., 2024). Positive and significant correlations were also obtained when data were normalised against nulls obtained by shuffling electrodes across epochs (rather than shuffling the order of electrodes within the epoch), which preserves variations in relative alpha power between electrodes in the null data, restricting tests to the spatiotemporal patterns that indicate travelling waves in the real data. Furthermore, we examined whether our results might be simply driven by differences in alpha power. To test this, we z-score transformed each electrode’s time series separately prior to the 2D-FFT. This normalises for differences in amplitude between electrodes and between individuals, controlling for potential differences in alpha power, while preserving the spatial properties of the cortical travelling waves (since the phase angles between the electrodes are preserved by this transform). The full results of this analysis are also reported in our Supplementary Materials; in brief, the same pattern of results and significant effects was present as per our primary analyses reported above, suggesting the effects were driven by variation in travelling wave strength, rather than simply a relationship between alpha power and agreeableness.

The analyses of lateral travelling waves reported thus far do not reveal whether the waves travel within the right hemisphere (from the temporal region towards the midline), or whether they travel between hemispheres. To address this, we performed 2D-FFTs including only selected electrodes: only right hemisphere electrodes, only electrodes closer to the midline, or the full set of nine potential central electrodes. Relationships between Agreeableness and travelling waves restricted to the right hemisphere were not significant (all *p* > 0.10), suggesting our results were not driven by waves travelling within the right hemisphere. Similarly, when our 2D-FFT was restricted to the more midline electrodes (from C3 to C4), no significant correlations were present (all *p* > 0.10). Only when we included electrodes from C5 to C6 or all 9 central line electrodes did significant effects become apparent (*rho* > 0.164, *p* < 0.005, *BF10* > 3.800). This suggests that the relationship between right to left cortical travelling waves and agreeableness/compassion is produced by interhemispheric travelling wave patterns rather than travelling waves from lateral to midline electrodes.

Finally, we tested for a correlation between agreeableness and the wave direction dominance ratio of right to left travelling wave patterns (within the 9–10 Hz temporal frequencies). This test did not show a significant relationship (*rho* = 0.097, *p* = 0.096, *BF10* = 0.280). In contrast, the correlation between agreeableness and the directional wave intensity of the average right to left travelling wave was significant (*rho* = 0.215, *p* < 0.001, *BF10* = 46.782). However, Hittner et al.’s (2003) test for the difference between dependent correlations with overlapping variables fell just short of formal significance (*z* = 1.533, *p* = 0.063). Interestingly, our results also indicated that the wave direction dominance ratio and the directional wave intensity were not related to each other (*rho* = 0.083, *p* = 0.153, *BF10* = 0.120). Overall, these results suggest that the right to left travelling wave strength is related to agreeableness, but that the rate of occurrence of this travelling wave pattern may not be related to agreeableness.

### 3.4 Openness/intellect relates to midline backwards travelling waves

**Fig. 5.**
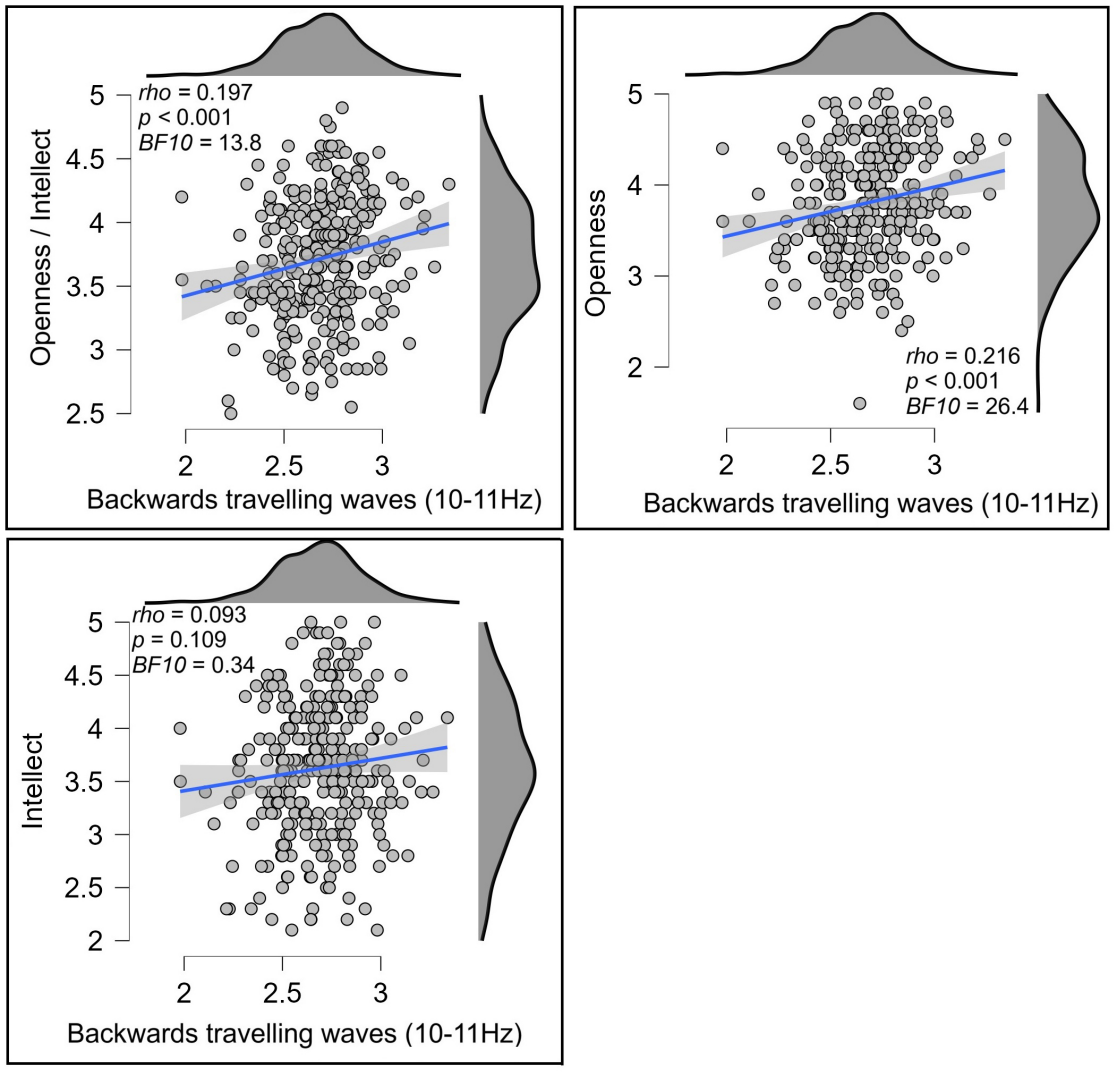
Scatterplots showing the relationship between Openness/Intellect (top left), openness (top right), or intellect (bottom left) and backwards travelling alpha waves (at 9 to 10 Hz, computed using a 2D-FFT from the midline electrodes, units in decibels) within the eyes open resting data. The blue lines indicate the linear regression line with the shaded area indicating the 95% confidence interval for this regression line.

Our 2D-FFT restricted to the midline electrodes (POz, Pz, CPz, Cz, FCz, Fz, AFz) showed a significant positive correlation between Openness/Intellect and backwards travelling waves at the lowest spatial frequency and 10–11 Hz in the temporal frequency (*rho* = 0.197, *p* < 0.001, *BF10* = 13.800, Fig. 5). To test the potential replicability of this result, we randomly split participants into separate groups of half the total sample size 10 separate times, then tested Spearman’s correlations on each of these reduced samples. Nine of the ten reduced samples showed a significant correlation (all *p* < 0.054, 9/10 *p* < 0.030, mean *p* = 0.022, all *rho* fell between 0.159 and 0.274, mean *rho* = 0.197). Given the weaker correlation strength than for relationships to Agreeableness, we suspected the fact that significant effects were present for only 9/10 splits might be due to a smaller sample size in the half sample splits. To assess this, we instead tested whether the correlations would remain significant if we used 75% of the sample in our splits. All of these ten reduced samples showed a significant correlation (all *p* < 0.036, mean *p* = 0.009, all *rho* fell between 0.141 and 0.224, mean *rho* = 0.194).

**Fig. 6.**
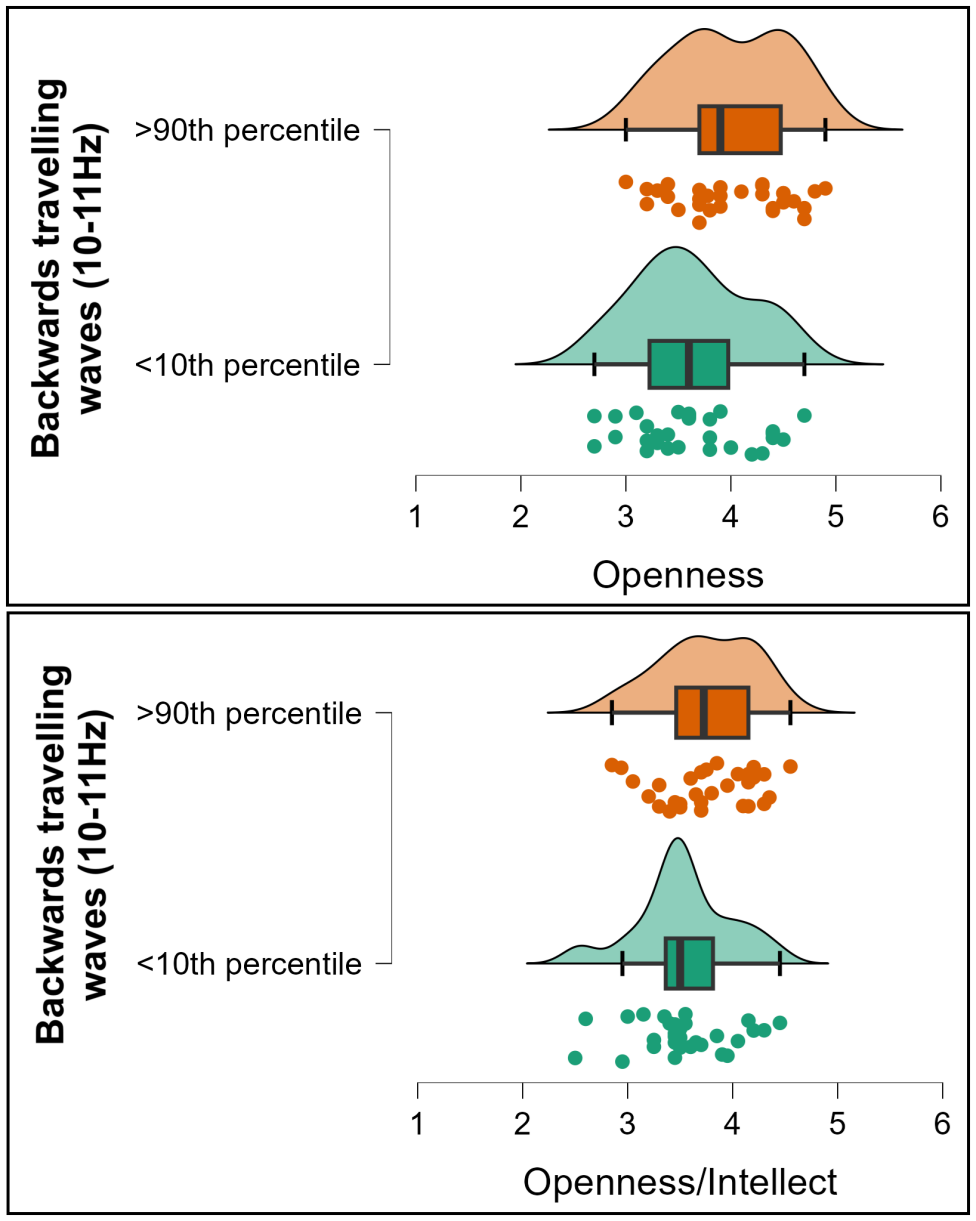
Raincloud plots depicting participants from the top and bottom 10^th^ percentile of backwards travelling wave strengths (10–11 Hz) and their associated scores for Openness (top) and Openness/Intellect (bottom).

In exploring whether the effect was related to specific aspects of Openness/Intellect, we found that the correlation was stronger for the Openness aspect (*rho* = 0.216, *p* < 0.001, *BF10* = 26.444, Fig. 5), and that the correlation with the Intellect aspect was not significant (*rho* = 0.093, *p* = 0.109, *BF10* = 0.344, Fig. 5); the difference between these correlations fell short of formal significance, *z* = 1.768, *p* = 0.077. Exploring further, a linear regression including these two variables as predictors for the backwards cortical travelling wave strengths revealed a significant effect for openness (*t* = 3.128, *p* = 0.002) but not for intellect (*t* = 0.956, *p* = 0.340), suggesting the effect was unique to openness. To illustrate the strength of the relationships between cortical travelling waves and openness/intellect, we extracted groups of participants showing the top 10% and bottom 10% of right to left travelling wave strength. The mean openness score from participants showing the top 10% of right to left travelling wave strength was 10.3% higher than the mean from the bottom 10%, and the mean openness/intellect score was 6.2% higher (see Fig. 6).

Furthermore, in addition to the relationship between backwards travelling waves and Openness/Intellect in the eyes open data, we tested for relationships to the eyes closed resting data to determine the consistency of the effect across different states. The same correlations between travelling waves and Openness/Intellect and the Openness aspect were significant in the eyes closed data, although with only weak Bayesian evidence in support of the relationship to Openness/Intellect (reported in full in the Supplementary Materials). These results indicate that the patterns were consistent across different resting states.

Finally, we tested for a correlation between openness/intellect and the wave direction dominance ratio for the backwards travelling waves (within the 10-11 Hz temporal frequencies). This test showed a significant relationship (*rho* = 0.161, *p* = 0.006, *BF10* = 13.556). The correlation between openness/intellect and the directional wave intensity for backwards travelling waves was also significant, but with a smaller effect size, with weak Bayesian evidence against the relationship (*rho* = 0.132, *p* = 0.024, *BF10* = 0.748). Hittner et al.’s (2003) test for the difference between dependent correlations with overlapping variables was not significant (*z* = 0.373, *p* = 0.355). Interestingly, our results also indicated that the wave direction dominance ratio and the directional wave intensity for the backwards waves were not related to each other (*rho* = 0.091, *p* = 0.117, *BF10* = 0.171). Overall, these results suggest that both the wave direction dominance ratio and directional wave intensity of backwards travelling waves were independently related to openness/intellect.

## 4 Discussion

In this study, we used a novel 3D-FFT analysis to determine whether any personality traits were related to the strength of cortical travelling waves, in both forwards/backwards and lateral directions. Our findings showed that agreeableness was related to the strength of interhemispheric alpha waves travelling from electrodes over the right temporal regions, and that this relation was largely attributable to the compassion (rather than politeness) aspect of agreeableness. Our findings also showed that openness/intellect was related to backwards travelling alpha waves down midline electrodes (travelling from anterior to posterior electrodes), and this relation was significant for openness but not intellect. The strength of the effect sizes of these relationships could be considered typical to relatively large when compared to empirically derived correlational effect size strength interpretations (equivalent to the 50^th^ to 75^th^ percentile of meta-analytically derived correlation values across a sample of 708 meta-analytic effect sizes (Gignac & Szodorai, 2016)). These relationships are also relatively strong compared to those commonly reported in the personality neuroscience literature using sufficiently powered sample sizes (e.g. Kuper et al., 2019; Pacheco et al., 2024), with strong Bayesian evidence for the relationship between openness/intellect and backwards travelling waves, and decisive Bayesian evidence for the relationships to agreeableness/compassion (Jeffreys, 1998). Furthermore, validation analyses involving randomly excluding 25 or 50% of our sample were consistent in showing a relationship between agreeableness and rightwards travelling waves, and 19/20 of these sub-sample tests showed a significant relationship between openness/intellect and backwards travelling waves. This demonstrates the effects were not dependent on a specific selection of participants, indicating our findings would likely replicate in independent samples. The strength and novelty of our results provides important new insights into potential neural mechanisms underlying personality traits.

Our findings build on previous research showing links between personality traits and neural oscillations (Pacheco et al., 2024). However, that previous research used methods that do not indicate whether oscillations are travelling. Although links between personality traits and standing oscillations are aligned with views that personality differences must be underpinned by stable differences in neural activity (Allen & DeYoung, 2017), they shed little light on the mechanistic reasons for neural correlates of personality traits. Our current findings extend this research to demonstrate relationships between specific personality traits and the strength of alpha travelling waves in specific directions. In particular, individuals scoring higher in openness/intellect (particularly in the openness aspect) showed higher values for backwards travelling waves from frontal to posterior midline electrodes, while individuals who scored higher in agreeableness (particularly in the compassion aspect) showed stronger rightwards travelling interhemispheric waves along the central electrode line. These findings align with other research that suggests measures of connectivity between brain regions might be particularly informative in personality neuroscience, and our results show broad consistency with functional magnetic resonance imaging (fMRI) connectivity patterns reported in previous research (Adelstein et al., 2011). The strength of the relationships we detected is also comparable to or stronger than much of the personality neuroscience field, in which robust sample sizes or meta-analyses typically provide *r*-values of 0.2 or less (DeYoung et al., 2025; Kuper et al., 2019; Pacheco et al., 2024). This means that the strength of the relationship we detected to compassion may indicate that cortical travelling waves reflect a particularly important marker for helping us understand variation in this trait.

The travelling wave patterns we assessed have been shown to provide an index of information flow through the cortex (Bailey, Hill, Godfrey, Perera, Hohwy, et al., 2024; Lozano-Soldevilla & VanRullen, 2019; Mohan et al., 2024; Muller et al., 2014; Pang et al., 2020). Specifically, travelling waves have been suggested to reflect propagation of prediction errors through the cortex and top-down prediction weighting from higher levels in the cortical hierarchy to lower levels (Alamia et al., 2023; Tarasi et al., 2025). Alpha travelling waves have also been shown to inhibit gamma activity and neuronal spiking, with the inhibition functioning to suppress predicted signals across distant cortical regions, perhaps explaining the mechanism by which travelling waves fulfil predictive processing functions (Xiong et al., 2024). Macro-scale alpha travelling waves (as detected in our study) have been shown to modulate micro-scale travelling waves, which, in turn, synchronise neuronal spiking (Sreekumar et al., 2020). This suggests that travelling waves provide a role in synchronising information processing across the cortex. Although our study design does not allow for causal inferences, the suggested functional role of cortical travelling waves seems more likely to provide a potential mechanism that may underpin variations in personality traits than that differences in cortical travelling waves in resting EEG data are downstream of differences in personality traits. Our findings may, therefore, suggest the relative strength of information flow between certain cortical regions relates to specific personality traits. Interpreting the location and direction of travelling wave patterns that relate to personality traits through this lens, coupled with prior evidence for the specialisation of brain regions, may offer unique insight into the neural mechanisms of personality differences.

Through this lens, it is intriguing that Agreeableness (especially compassion) relates to travelling waves propagating from the right to the left hemisphere, with an origin detected in electrodes over temporal and temporoparietal brain regions. Agreeableness is characterised by altruism, empathy, and cooperation, and associated with social cognition and perspective taking (Graziano et al., 2007; Nettle & Liddle, 2008). Research indicates the superior temporal region is associated with interpreting the actions and intentions of others based on their movements (Pelphrey & Morris, 2006). In alignment with this, research using fMRI has shown that agreeableness correlates with volumes in the left superior temporal region (Li et al., 2017, but cf, Allen et al., 2017). Activity in the right temporoparietal junction has been shown to be associated with the processing of empathy, sympathy, taking another’s perspective, and processing social cues (Decety & Lamm, 2007). fMRI research also indicates that connectivity to the right temporoparietal junction is predictive of agreeableness for a majority of individuals (Nostro et al., 2018). Furthermore, myelin-based microstructural connectivity in an interhemispheric network including the right temporoparietal junction has been shown to both relate to agreeableness and mediate the better life satisfaction associated with agreeableness (Wu et al., 2024). Our data did not enable accurate source analysis and source analyses of travelling waves face important limitations that may prohibit detection of the relationships we report in our scalp space analyses (Alamia et al., 2020). However, given the effects of volume conduction on EEG, this means we cannot draw conclusions about the sources of the travelling waves. Nevertheless, the consistency between functional associations reported for relevant brain regions and our findings of stronger travelling waves from electrodes over these regions in people high in trait compassion provides an intuitive link between established neuroanatomic specialisation and a personality trait. This consistency suggests a potential mechanistic pathway between neural activity, behaviour, and personality traits. Although we note that causal inferences are not possible with our study design, we view this pathway as a plausible account of our descriptive findings.

Travelling wave patterns may originate via both genetic and environmental influences. Individuals who inherit genes that influence their brain activity towards stronger propagation of activity from certain brain regions may be more commonly motivated by consideration of others, resulting in an increased frequency or intensity of thoughts, emotions, and behaviours related to compassion. Individuals who experience life events that cause them to be motivated by compassionate concern for others may also develop more synaptic connections from these brain regions, leading to stronger travelling waves from those regions, perhaps driving compassionate behaviour in the future. Both pathways likely interact, with different degrees of influence within different individuals, leading to higher endorsement of higher scores on compassion questions when answering a personality questionnaire. Individuals high in agreeableness may then have an increased proclivity to rest in a state that engages rightwards travelling waves and relevant networks, in alignment with suggestions that personality traits correspond to probability distributions of mental states (Fleeson & Gallagher, 2009). Alternatively (or additionally), the differences may be enduring, and may influence behaviour on a daily basis, in alignment with more biologically oriented theories of personality (e.g. Depue & Morrone-Strupinsky, 2005). In support of this, travelling wave directions have been shown to relate to structural connectivity gradients in the human connectome detected by MRI (Koller et al., 2024), suggesting the travelling wave patterns we detected may reflect traits persisting outside of the resting period. Additionally, although the differences in correlation strengths did not quite reach formal significance, our exploratory analyses suggested the relationship between travelling waves and agreeableness may be stronger for travelling wave strengths than for how often the travelling waves occurred. This may indicate a consistent influence of travelling waves from the right hemisphere on this personality trait, which may reflect a constantly higher background strength of the contribution from the generating sources of these rightwards travelling waves over cognition in individuals who scored higher in agreeableness (rather than a higher frequency of occurrence of this influence).

Turning now to openness/intellect, individuals higher on this trait are characterised by abstract thinking, cognitive flexibility, and curiosity (DeYoung et al., 2014; Kaufman et al., 2016). Interestingly, our analyses indicated that backwards travelling waves were associated with openness, but not intellect. While openness describes engagement in perception, fantasy, aesthetics, and emotion, intellect reflects engagement with abstract and semantic information via reasoning (DeYoung, 2015). Backwards travelling waves have previously been reported to indicate the voluntary control of attention (Alamia & VanRullen, 2019; Pang et al., 2020), an executive function that relates to the intellect aspect of openness/intellect rather than openness (Smillie et al., 2016). However, research examining travelling waves in mindfulness meditators suggests that during resting, backwards waves might reflect thoughts about the future or past, or daydreaming, rather than the executive functions required by cognitive tasks (Bailey, Hill, Godfrey, Perera, Hohwy, et al., 2024). In this context, the stronger backwards wave strength in individuals high in trait openness might reflect engagement of daydreaming during resting EEG recordings, associated with stronger backwards waves, but reflective of top-down processes that are not indicative of executive functions associated with the intellect aspect of openness/intellect. This pattern of results aligns with fMRI work showing that openness is related to the efficiency of activation of the default mode network (Beaty et al., 2016), as well as connectivity to core midline hubs of the default mode network (Adelstein et al., 2011)—brain regions associated with cognitive flexibility, creativity, and imagination. Furthermore, our exploration of whether the relationship between backwards travelling waves and openness/intellect was driven by the proportion of occurrence of backwards waves or their strength indicated that relationships were significant for both their occurrence and their strength. An interesting side note here is that the rate of occurrence of these waves and their strengths were not significantly correlated (when their strength was measured independently of the influence of occurrence rates). This suggests that although travelling wave strengths and occurrence rates were independent of each other, both were related to the personality trait of openness/intellect in the same direction. This consistency provides additional support for the assertion that travelling wave strengths are likely to reflect engagement of cognitive processes that are relevant to personality traits. Both more frequent activations of the backwards travelling waves and stronger activations likely reflect increased engagement of relevant cognitive processes associated with openness/intellect. The indication that travelling wave strengths and rates of occurrence are independent of each other may also be interesting to explore in future research into the function and mechanisms of travelling waves.

While the implications of our results are predominantly focused on our understanding of neural sources of personality traits, our results have potential practical applications for personality change interventions. Some estimates suggest around two thirds of people would like to change some aspect of their personality—a finding reflected in the prominence of the self-improvement industry (Miller et al., 2019). There is also growing evidence that interventions can change personality traits (Bleidorn et al., 2019). Recent proof-of-concept research suggests that cortical travelling waves can be modulated by modified transcranial alternating current stimulation (Lee et al., 2023). Future research might therefore explore brain stimulation methods targeting cortical travelling waves as a potential avenue for increasing trait levels of compassion or openness, for individuals seeking to do so.

Our findings indicated decisive Bayesian evidence for a relationship between agreeableness/compassion and rightwards travelling waves, and strong evidence for a relationship between openness and backwards travelling waves. Nonetheless, our conclusions are limited by several considerations. First, our study was exploratory in nature, using a data driven approach, rather than hypothesis driven. This is a strength in one sense, enabling detection of relationships unlikely to be conceived via hypothesis driven approaches (Bailey, Fulcher, et al., 2024). Additionally, our tests applied extensive multiple comparison controls, and our results were robust to random splits of the data into subsamples. More specifically, we showed the same significant patterns even when random subsets of participants from our large sample were selected for testing. This method eliminates sampling biases as a potential explanation for our results, and suggests our findings reflect real effects. However, previous research with robust multiple comparison controls, large samples, and effects that are consistent across random within sample splits still at times does not replicate (e.g., Bailey et al., 2019, 2021; Dinga et al., 2019; Drysdale et al., 2017; Fruehlinger et al., 2024; Jach et al., 2020). Hypothesis-driven testing in an independent dataset would provide increased confidence that our results reflect a true pattern. It is also important to acknowledge that our data primarily have descriptive rather than explanatory value; it shows that personality traits are associated with cortical travelling waves, but cannot confirm or refute any explanation for such associations. Of course, since this is (to our knowledge) the first study examining personality in relation to travelling waves, descriptions of any such associations are required before hypotheses regarding their underlying mechanisms can be developed and tested. Further, we note that although the effect sizes we detected are in the typical to relatively large range relative to empirically derived correlational effect size interpretations, they only explain 0.047 to 0.081% of the variance (for openness to experience and compassion, respectively).

Second, previous research on our data indicates that agreeableness can be decoded from spatially stationary non-oscillatory activity (which shows a 1/f log-power log-frequency distribution) after excluding oscillatory activity (Pacheco et al., 2024). Additionally, the same research showed measures of standing oscillatory activity alone (after removing the influence of non-oscillatory 1/f activity) provided better decoding of activity related to personality traits than measures of broadband activity (Pacheco et al., 2024). This finding of increased sensitivity after separating oscillatory and non-oscillatory activity is common across different populations (e.g. McQueen et al., 2024) highlighting the potential importance of addressing 1/f non-oscillatory activity.

While the 2D and 3D-FFTs we used allowed novel measurement of cortical travelling waves in multiple spatial directions, FFT analyses (including 2D-FFT and 3D-FFT analyses) do not separate oscillatory activity from non-oscillatory activity. In the temporal domain, neural activity has been shown to contain oscillatory activity, which exhibits a repeating rhythm of peaks and troughs, as well as non-oscillatory activity, which is composed of shifts in voltage without repeating cycles, but which, nevertheless, contributes power across a range of frequencies when EEG data are analysed using FFT methods (Donoghue et al., 2020). Without applying methods to separate oscillatory and non-oscillatory activity, our results cannot indicate whether the patterns we observed are driven by travelling waves that oscillate (temporally), or simply frequency voltage shifts that overlap with the alpha band frequencies and travel across the scalp, without exhibiting true repeating oscillatory cycles in the temporal domain. Future research might address this using travelling wave methods that control for non-oscillatory activity, but we are currently not aware of such methods that can be applied to travelling wave analyses. However, it is also worth mentioning that there is currently no indication that the measurement of alpha travelling waves are adversely affected by non-oscillatory activity. Alpha oscillations also show the largest peak above non-oscillatory activity, and previous research shows that when analyses are restricted to alpha peaks above non-oscillatory activity, travelling waves are still prominent (Zhang et al., 2018). Furthermore, modelling and empirical work has suggested that unless neurophysiological effects involve differences in synaptic currents decay times, correcting for the non-oscillatory activity can introduce larger measurement errors than the potential gains in measurement accuracy (Brake et al., 2024).

Finally, the data used in the current analysis were recruited from a WEIRD population (white, educated, industrialised, rich, and democratic) (Pacheco et al., 2024). As such, it is not clear that our results would generalise to other populations. Future research in other populations is required to determine whether the relationships between personality traits and cortical travelling waves in different cultures are in alignment with calls for the future of EEG research to be more inclusive and representative (Mushtaq et al., 2024).

## Ethics

Ethics approval was provided by the Human Research Ethics Committees of The University of Melbourne. All participants provided written informed consent prior to participation in the study.

## Supplementary Material

Supplementary Material

## Data Availability

The cortical travelling wave data and code associated with this study can be obtained from the project repository on the Open Science Framework (https://osf.io/dn4th/).
