# Supplementary material for: Cortical travelling waves relate to variation in personality traits
Source: Imaging Neurosci (Camb). 2025 Aug 20;3:IMAG.a.119. doi: 10.1162/IMAG.a.119 (PMC12368610; doi:10.1162/IMAG.a.119)
Supplement: Supplementary Material [file IMAG.a.119_supp.pdf]

<u>Cortical travelling waves relate to variation in personality traits</u>

Neil W Bailey, Luiza Bonfim Pacheco, Luke D. Smillie

Supplementary Materials

Right to left interhemispheric travelling waves were present in our data

Our one-sample t-tests showed that the right to left interhemispheric travelling waves across 8-13Hz in our real data were significantly stronger than the surrogate distributions, $t(295) = 102.886$, $p < 0.001$, Cohen's $d = 5.980$, $BF10 = 7.049*10^{228}$ (mean = 71.306, SD = 11.757). These values suggest that the values for rightwards travelling waves were on average >70 times stronger than would be expected if there were no rightwards travelling wave signals present in the data, with Bayesian evidence that indicates essentially certain confidence in the presence of these travelling waves. Furthermore, no individual participant showed a value below 40, indicating that all participants showed right to left travelling wave strengths that substantially exceeded their surrogate distribution. Similarly, our one-sample t-tests showed that the backwards travelling waves at 8-13Hz exceeded our surrogate values, by an even larger amount than the right to left travelling waves $t(295) = 227.272$, $p < 0.001$, Cohen's $d = 13.210$, $BF10$ = infinite (mean = 177.273, SD = 13.344). These values suggest that the real backwards travelling waves were on average > 175 times stronger than would be expected if there were no backwards travelling wave signals present in the data, with Bayesian evidence for the presence of backwards travelling waves that was stronger the capacity for JASP to measure. Furthermore, no individual participant showed a backwards travelling wave value below 140, indicating that all participants showed backwards travelling wave strengths that substantially exceeded their surrogate distribution.

A left-to-right travelling wave may also correlate with agreeableness

In addition to the significant rightwards travelling wave cluster in our cluster-based statistical analysis performed the 3D-FFT outputs, a cluster from 8-9Hz travelling left to right at the maximal spatial frequency showed a similar positive correlation with Agreeableness. This cluster also showed a similar positive correlation with Agreeableness when examined in the 2D analysis along the central electrodes, although with Bayesian evidence against the alternative hypothesis ($rho = 0.155$, $p = 0.007$, $BF10 = 0.718$). The significant effect was also present for compassion ($rho = 0.158$, $p = 0.006$, $BF10 = 0.608$), but not politeness ($rho = 0.106$, $p = 0.068$, $BF10 = 0.215$); the difference between these effects was not significant, $z = 0.823$, $p = 0.411$. Further, a linear regression including compassion and politeness as predictors for the leftwards travelling wave strengths did not yield a significant effect for either compassion ($t = 1.629$, $p = 0.104$) or politeness ($t = 0.744$, $p = 0.458$). A depiction of the pattern of these rightwards travelling waves is presented in Figure S1.

Eyes-closed data showed the same relationships as eyes-open data

In addition to the relationship between right to left travelling waves and Agreeableness in the eyes open data, we tested for a relationship in the eyes closed resting data to determine the consistency of the effect across different states. The correlation between Agreeableness and eyes closed resting right to left traveling waves at the highest spatial frequency and 9-10Hz was significant (rho = 0.236, p < 0.001, BF10 = 7.467). The relationship was also significant for

compassion (rho = 0.246, p < 0.001, BF10 = 14.862) and for politeness, although with Bayesian evidence against the effect (rho = 0.147, p = 0.011, BF10 = 0.324). A linear regression including compassion and politeness as predictors for the rightwards cortical travelling wave strengths in the eyes-closed resting data was also undertaken. This analysis showed a significant effect for compassion (t = 2.841, p = 0.005) but not for politeness (t = 0.535, p = 0.593), suggesting the effect was specific to compassion, similar to our analysis of the eyes open resting variables. This result indicates that the patterns are consistent across different resting states.

Similarly, in addition to the relationship between backwards travelling waves and Openness/Intellect in the eyes open data, we tested for a relationship in the eyes closed resting data. The correlation between Openness/Intellect and eyes closed resting backwards traveling midline waves at the lowest spatial frequency and 10-11Hz was significant, although with only weak Bayesian evidence in support of the relationship (rho = 0.157, p = 0.007, BF10 = 1.766). The relationship was also significant for openness (rho = 0.196, p < 0.001, BF10 = 23.096), but not for intellect, with Bayesian evidence against the effect (rho = 0.055, p = 0.349, BF10 = 0.096). This result indicates that the patterns were consistent across different resting states.

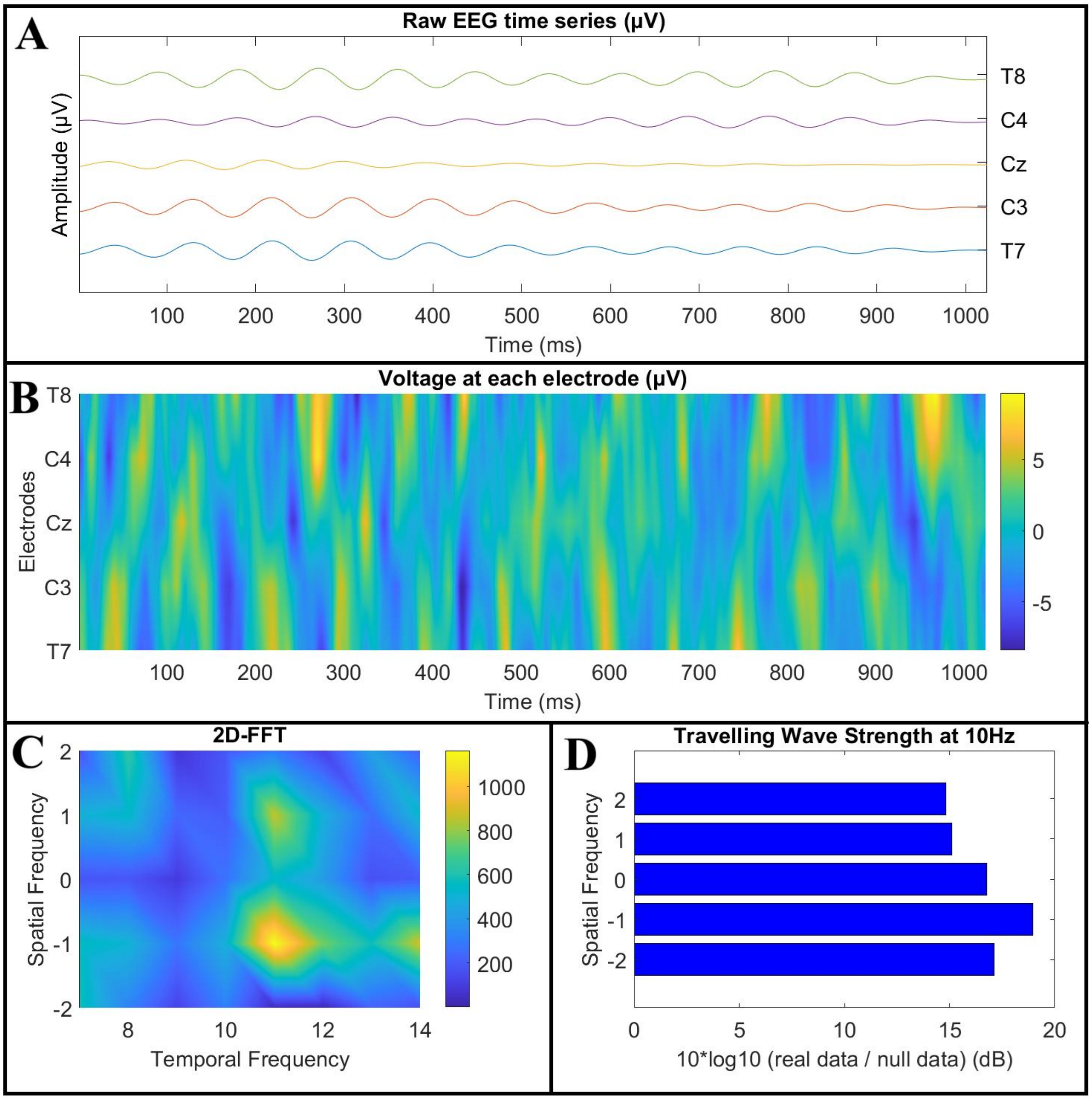

Figure S1. A visual representation of the travelling wave computation method using a 2-dimensional fast Fourier transform (2D-FFT) for the right to left travelling waves along central electrodes. A/B) First, 1 second epochs were extracted from the continuous electroencephalography (EEG) data after removal of artifacts. The time-series from each electrode were then organised into matrices, with posterior electrodes at the bottom of the matrices and anterior electrodes at the top (and left electrodes on the left side of the 3D matrices, and right electrodes on the right side), as depicted in A and B (after applying a bandpass filter in the alpha frequencies of interest to highlight the travelling waves). C) Each epoch was then used as the input for a 3D-FFT (MATLAB), or 2D-FFT in the case of our post-hoc tests (as depicted above for simplification of the visualisation). This provided a matrix providing power values in each spatial and temporal frequency. Within this matrix, the x-axis provides power values at each temporal frequency, while the y-axis provides power values at each spatial frequency – shown in C with values above 0 reflecting backwards travelling spatial waves (from frontal to posterior electrodes), values below 0 reflecting forwards travelling spatial waves (from posterior to frontal electrodes), and values at 0 reflecting standing waves (waves that do not travel). In our 3D-FFT (not depicted in this figure), the z-axis would reflect lateral travelling waves (from left to right or right to left). D) Finally, to assess the travelling wave strength in each spatial and temporal frequency, we divided the power in each cell in the matrix by the power obtained after randomly shuffling the data 100 times, repeating the FFT computations for each shuffle, and averaging the outputs of these null FFTs. In the figure above, the random shuffles were obtained by shuffling electrodes between epochs, as per the analysis performed in some of our post-hoc validation tests. However, within our primary 3D-FFT tests, we shuffled the electrode order within each epoch. We then computed a log10 transform on the output of this division and multiplied this value by 10 to obtain a measure of the strength by which the real travelling wave strength exceeded the permutation-based null travelling wave strength, on the decibel scale (Figure 1D). Note that the lateral travelling waves were weaker in strength than the forwards/backwards travelling waves (which can be seen when comparing the current Figure to to Figure 1 in the main manuscript).

Analysis of latent personality variables showed the same patterns as our primary analyses

Next, to assess the robustness of our results to different analysis methods, we tested relationships between the cortical travelling waves that showed significant relationships to personality traits in our primary analyses to latent personality factors. The same pattern of associations between rightwards travelling waves and Agreeableness (and its aspects) was evident when personality traits were estimated as latent variables: Waves travelling from the right to the left along the C line were significantly correlated with latent Agreeableness (rho = 0.283, $p < 0.001$, BF10 = 1317.217). A similar correlation was present with latent compassion (rho = 0.270, $p < 0.001$, BF10 = 436.566), which was stronger than the effect for latent politeness (rho = 0.183, p = 0.002, BF10 = 2.112). A linear regression including latent compassion and latent politeness as predictors for the rightwards cortical travelling wave strengths was also undertaken. This analysis showed a significant effect for compassion (t = 3.466, $p < 0.001$) but not for politeness (t = 1.036, p = 0.301), suggesting the effect was specific to latent compassion, similar to our analysis of the non-latent variables.

The patterns noted for Openness/Intellect were also similar for the latent variables, but weaker: latent Openness/Intellect was positively correlated with backwards travelling wave strength (rho = 0.137, p = 0.018). The same correlation was present for the latent variable of Openness (rho = 0.171, p = 0.003), but again, not for the latent variable of Intellect (rho = 0.088, p = 0.131);

the difference between these correlations was statistically non-significant, $z = 1.186$, p = 0.236. A linear regression including these two variables as predictors for the backwards cortical travelling wave strengths confirmed a significant effect only for openness (t = 2.715, p = 0.007) but not for intellect (t = 0.954, p = 0.341).

The centroparietal and frontocentral electrode lines also showed similar relationships to agreeableness

The relationship between Agreeableness and rightwards travelling waves was also significant when our 2D-FFT was computed using the fronto-central, and centroparietal lateral electrode lines. Waves travelling from the right to the left at the maximum spatial frequency along the centroparietal electrode line were significantly correlated with Agreeableness (rho = 0.214, p < 0.001, BF10 = 21.592). A similar correlation was present with compassion (rho = 0.224, p < 0.001, BF10 = 42.512), and with politeness (although weaker, with Bayesian evidence against the relationship: rho = 0.134, p = 0.021, BF10 = 0.502). A linear regression including compassion and politeness as predictors for the rightwards cortical travelling wave strengths was also undertaken. This analysis showed a significant effect for compassion (t = 3.083, p = 0.002) but not for politeness (t = 0.671, p = 0.503), suggesting the effect was specific to compassion, similar to our analysis of the non-latent variables.

The correlations were also significant when testing relationships in the fronto-central electrode line, although of weaker strength and with lower Bayesian evidence than for the central and centroparietal lines. Waves travelling from the right to the left along the FC line were significantly correlated with Agreeableness (rho = 0.204, p < 0.001, BF10 = 3.537). A similar correlation was present with compassion (rho = 0.204, p = 0.011, BF10 = 1.864), and with politeness (although weaker: rho = 0.140, p = 0.016, BF10 = 0.608). A linear regression including latent compassion and latent politeness as predictors for the rightwards cortical travelling wave strengths was also undertaken. This analysis showed a non-significant trend towards a significant effect for compassion (t = 1.919, p = 0.056) but not for politeness (t = 1.189, p = 0.235).

Using the mean instead of the 75th percentile showed the same relationships

Additionally, despite our rationale that testing the 75th percentile of travelling wave strengths across epochs would be more likely to enable detection of relationships to personality traits due to these epochs representing stronger engagement of neural processes of interest, it is more typical to test the mean across epochs. To assess the consistency of our results when applying more typical travelling wave quantification methods, we repeated tests of relationships between personality traits and both our 3D-FFT and 2D-FFT outputs using mean values across epochs. These analyses showed the same pattern of results and significant effects as our primary analyses. As noted in our main manuscript, the 3D-FFT results recapitulated the results we detected when using the 75th percentile. When using the mean travelling wave strength (instead of the 75th percentile), a significant relationship between travelling waves and agreeableness was present ($p_{FDR} = 0.003$, p < 0.001), and a significant relationship between travelling waves and Openness/Intellect was present ($p_{FDR} = 0.031$, p = 0.012, see Figure S2). Within our 2D-FFT analyses, the correlation between Agreeableness and the mean eyes-open resting right to left traveling waves at the highest spatial frequency and 9-10Hz was significant (rho = 0.205, p < 0.001, BF10 = 15.433). The relationship was also significant for compassion (rho = 0.243, p < 0.001, BF10 = 141.583), but not for politeness, with Bayesian evidence against the effect (rho = 0.103, p = 0.076, BF10 = 0.208). A linear regression including compassion and

politeness as predictors for the mean rightwards cortical travelling wave strengths in the eyes-open resting data was also undertaken. This analysis showed a significant effect for compassion ($t = 3.659$, $p < 0.001$) but not for politeness ($t = -0.030$, $p = 0.976$), suggesting the effect was specific to compassion, similar to our analysis of the effects obtained by testing the values at the 75th percentile.

Furthermore, the correlation between openness/intellect and mean eyes-open resting backwards traveling waves at the lowest spatial frequency and 10-11Hz was significant (rho = 0.182, p = 0.002, BF10 = 5.794). The relationship was also significant for openness (rho = 0.220, p < 0.001, BF10 = 18.120), but not for intellect, with Bayesian evidence against the effect (rho = 0.079, p = 0.178, BF10 = 0.207). A linear regression including openness and intellect as predictors for the backwards cortical travelling wave strengths in the eyes-open resting data was also undertaken. This analysis showed a significant effect for openness ($t = 3.090$, $p = 0.002$) but not for intellect ($t = 0.652$, $p = 0.515$), suggesting the effect was specific to openness, similar to our analysis of the effects obtained by testing the values at the 75th percentile.

Finally, an exploratory analysis of correlations between travelling waves and personality traits indicated that correlation strengths were higher when extracting travelling waves from the epochs showing above median travelling wave strength, while correlation strengths obtained by extracting epochs that showed below median travelling wave strength diminished as the percentile of travelling wave strength across the epochs fell progressively lower (see Figure S3). This effect is in line with our expectation that periods when travelling waves were activated are likely to reflect the engagement of these neural mechanisms to perform cognitive processes, with the strength or likelihood of those cognitive processes then likely to relate to personality traits. Thus, our results provide additional support for our approach of using the 75th percentile as a measure that is likely to be more sensitive to relationships with personality traits.

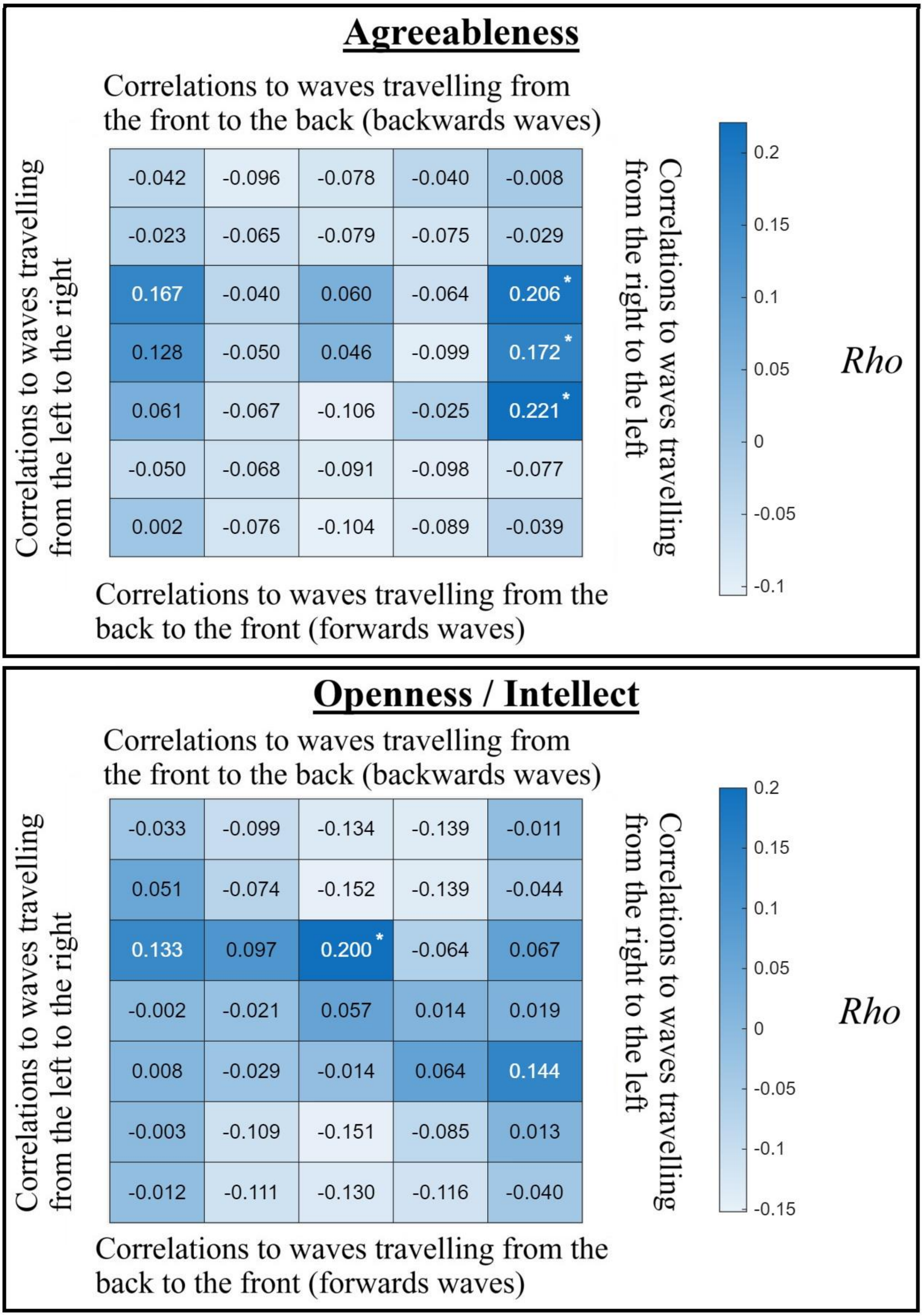

Figure S2. Spearman's correlations between mean 3D-FFT travelling wave values at each spatial frequency and direction, and at 10Hz in the frequency domain, and agreeableness (top) or openness / intellect (bottom). * indicates cells that were involved within a cluster that passed

our initial cluster statistical test of relationships between the personality traits and 3D-FFT outcomes ($p_{FDR} < 0.05$), with values in each cell representing travelling waves across the directions that could be measured across the scalp electrodes. Note that for openness/intellect, only one significant cell is depicted, as the other significant cells were found at 11Hz.

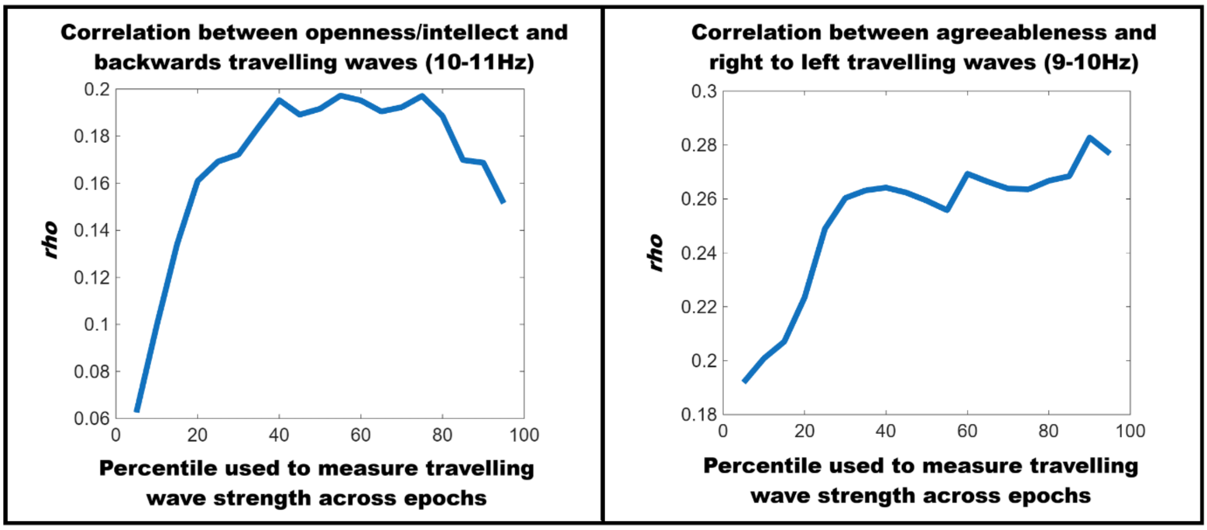

Figure S3. Correlation strengths between personality traits and travelling wave strength (selected from the significant temporal and spatial frequencies we observed in our primary analyses). The correlation strength (*rho*) is depicted after selecting different percentiles of travelling wave strengths across the epochs to obtain the travelling wave strength outcome measure. Note that the *rho* value is higher for percentiles above the median compared to below the median, in alignment with our rationale that periods when travelling wave mechanisms are engaged are more likely reflect engagement of cognitive processes that then relate to personality traits. This provides support for our analytical decision to extract travelling wave strengths at the 75th percentile across epochs rather than the mean.

Results for agreeableness were robust against different normalisation approaches

Despite these interesting results, inspection of the data revealed that the decibel values for the rightwards lateral travelling waves at the highest spatial frequency were typically negative after division by the mean of 100 null shuffles. This suggests that the null shuffles of electrode orders contained more power than the real data in these highest lateral spatial frequencies, requiring consideration of whether the positive correlations we detected were driven by the presence of true lateral travelling waves. This pattern might be explained by an increased strength of travelling waves between the midline electrodes compared to waves propagating from outer lateral electrodes, such that when the midline electrodes are “spread out” in the null shuffles of the data, the increased strength of travelling waves between midline electrodes is transferred to the higher spatial frequencies. As a result, the log transform of the real values divided by the null shuffle values produces negative values on the decibel scale in the normalised data. This prompted us to assess whether the positive correlations we detected were driven by the presence of true lateral travelling waves rather than somehow being driven by the normalisation procedure.

To assess whether our result in these highest lateral spatial frequency cells may have been driven by the normalisation procedure (which would suggest an effect driven by non-spatial patterns), we tested correlations between agreeableness, compassion, and the mean null shuffle versions of the data. If these correlations were significant, it would demonstrate that the normalisation against the null shuffles was driving the significant correlations we have reported for rightward travelling waves. The correlation between the null shuffle versions of the data and agreeableness was not significant (rho = 0.043, p = 0.463, BF10 = 0.110), and nor was the relationship to compassion (rho = 0.017, p = 0.775, BF10 = 0.077). To further assess whether our result in these highest lateral spatial frequency cells may have been influenced by the normalisation procedure, we tested correlations against agreeableness on the log10 transformed versions of the real data, after normalisation against stationary (1D-FFT) mean alpha power across the electrodes of interest (instead of the null shuffled versions of the electrode order) (Zeng et al., 2024). This provided control for variations in standing wave alpha power (in the temporal but not spatial domain), while still measuring the cortical travelling wave strength (Zeng et al., 2024). Within these tests, the correlation between agreeableness and power in the highest right to left travelling spatial frequency from 9-10Hz was still significant, (rho = 0.262, p < 0.001, BF10 = 611.413), as was the correlation with compassion (rho = 0.256, p < 0.001, BF10 = 150.596).

To further address this potential issue, we also tested for correlations after normalising data to null shuffles obtained by randomly swapping electrodes between epochs. This approach destroyed the spatiotemporal relationships that produced travelling waves, as the timing of oscillations between epochs would not be synchronised, but preserved both variations in alpha power, and variations in the relationship between electrodes in alpha power. These correlations were also significant - the correlation between agreeableness and power in the highest right to left travelling spatial frequency from 9-10Hz was still significant, (rho = 0.205, p < 0.001, BF10 = 48.979), as was the correlation with compassion (rho = 0.204, p < 0.001, BF10 = 21.371). Interestingly, normalising using this method demonstrated that the rightward travelling waves did exceed the null shuffled data in many participants, but not all participants, with a mean value of 0.170 dB (SD = 0.306, minimum = -0.786, maximum = 0.885). This suggests that on average, the real rightwards travelling wave strength exceeded the strength of the null shuffles, but not by much, and not for every participant. This pattern indicates that although the rightwards travelling wave does reflect a true signal in the data, the signal is weak and not present in all individuals. As such, these tests confirm that the effects were indeed driven by a relationship between true rightwards travelling wave strength. True rightwards travelling waves were present more commonly in individuals scoring higher in agreeableness, and individuals higher in agreeableness showed rightwards travelling waves that exceeded the values obtained via null shuffles of the data that destroyed the travelling wave patterns by a larger amount compared to individuals scoring lower in agreeableness and compassion.

Additionally, previous research on a subset of the dataset used in the current study has shown that agreeableness is negatively correlated with posterior alpha power (Jach et al., 2020). As such, we performed an additional test to assess the potential that our results might be simply driven by differences in alpha power. In this test, we z-score transformed each electrode’s time series separately prior to the 2D-FFT. This normalises for differences in amplitude between electrodes and between individuals, controlling for potential differences in alpha power, while preserving the spatial properties of the cortical travelling waves (since the phase angles between the electrodes are preserved by this transform). After these computations, the correlation between Agreeableness and travelling waves from the far right at 9-10Hz was still

significant, and in fact even stronger than the initial tests (rho = 0.274, $p < 0.001$, BF10 = 741.186). The correlation was also significant for compassion (rho = 0.295, $p < 0.001$, BF10 = 1268.632), and for politeness, although again weaker than for compassion (rho = 0.175, p = 0.003, BF10 = 2.302). This indicates that the relationships were present even after controlling for potential differences in alpha power across the electrodes.

Analyses of subsets of lateral electrodes indicates results for agreeableness were driven by interhemispheric travelling waves

Finally, we note that the analyses of lateral travelling waves reported thus far do not reveal whether the waves travel within each hemisphere, or whether they travel between hemispheres. To address this, we performed additional 2D-FFTs that 1) only included the right hemisphere central electrodes (T8 to Cz), 2) included midline electrodes only (C3 to C4), 3) included lateral electrodes, but not temporal electrodes (C5 to C6), and 4) included all central line electrodes (T7 to T8, 9 electrodes). Relationships between Agreeableness and lateral cortical travelling waves restricted to the right hemisphere only were not significant at any spatial frequency (all $p > 0.10$ when only electrodes from T8 to Cz were included in the 2D-FFT). This suggests that our primary results were not driven by waves travelling from the right-most electrodes to the midline. Similarly, when our 2D-FFT was restricted to electrodes from C3 to C4, no significant correlations were present at any spatial frequency (all $p > 0.10$). Only when we included electrodes from C5 to C6 did significant effects become apparent, although the effects within this analysis were weaker than our results from analyses that included T7 and T8 electrodes. The analysis including electrodes from C5 to C6 showed a significant correlation between Agreeableness and right to left travelling waves at the middle spatial frequency and 9-10Hz (rho = 0.165, p = 0.004, BF10 = 3.824). The analysis that included the 9 central line electrodes (T7 to T8) showed a significant correlation between agreeableness and right to left travelling waves at the second lowest spatial frequency (rho = 0.269, $p < 0.001$, BF10 = 540.882). Given that the list of electrodes from T8 to Cz and from C3 to C4 both contained five electrodes (the same number of lateral electrodes as in the 3D-FFT and our post-hoc 2D-FFTs), the lack of effect when testing the right hemisphere alone and midline electrodes is unlikely to be due to a reduced number of electrodes. Therefore, the pattern of results suggests that the relationship between right to left cortical travelling waves and agreeableness / compassion is produced by interhemispheric cortical travelling wave patterns rather than travelling waves from lateral electrodes to midline electrodes.

Do travelling waves and their relationships to personality reflect states or traits?

In considering the explanation for our results, we note that the EEG recording session itself may have acted as a conditioning stimulus, prompting certain thoughts, emotions, or states of mind, and that these factors may have interacted with personality traits to lead to the findings we have reported. If this is the case, our findings might not reflect stable processes underlying agreeableness or openness, but rather a proclivity towards specific mental states that are associated with specific neural travelling wave markers and elicited only within specific contexts—in this case, a neuroscientific study (Jach et al., 2020). However, even if this were the case, it would not negate the finding that compassion and openness were linked with travelling waves. Given the myriad of potential interacting influences on participants in the paradigm, no specific influence seems more likely than the interpretation that our results reflect a neural marker of the two personality traits (Jach et al., 2020). Additionally, travelling wave directions have been shown to follow structural connectivity gradients (Koller et al., 2024), suggesting the travelling wave patterns we detected may reflect traits that persist outside of the resting period.

Future research might explore whether stronger travelling waves observed at rest are related to stronger travelling waves during tasks, which might indicate that resting travelling wave strengths are trait markers that have an influence on behaviour.

Supplementary Materials References

Jach, H. K., Feuerriegel, D., & Smillie, L. D. (2020). Decoding personality trait measures from resting EEG: An exploratory report. *Cortex*, *130*, 158-171.

Koller, D. P., Schirner, M., & Ritter, P. (2024). Human connectome topology directs cortical traveling waves and shapes frequency gradients. *Nature Communications*, *15*(1), 3570.
